# In situ development of a methanotrophic microbiome in deep-sea sediments

**DOI:** 10.1038/s41396-018-0263-1

**Published:** 2018-08-28

**Authors:** S. E. Ruff, J. Felden, H. R. Gruber-Vodicka, Y. Marcon, K. Knittel, A. Ramette, A. Boetius

**Affiliations:** 1 Max Planck Institute for Marine Microbiology, Bremen, Germany; 2 MARUM Center for Marine Environmental Sciences, University of Bremen, Bremen, Germany; 3 Alfred Wegener Institute, Helmholtz Center for Polar and Marine Research, Bremerhaven, Germany; 4 Present Address: Department of Geoscience, University of Calgary, Calgary, AB Canada; 5 Present Address: Institute for Infectious Diseases, University of Bern, Bern, Switzerland

**Keywords:** Microbial ecology, Biogeochemistry, Food webs

## Abstract

Emission of the greenhouse gas methane from the seabed is globally controlled by marine aerobic and anaerobic methanotrophs gaining energy via methane oxidation. However, the processes involved in the assembly and dynamics of methanotrophic populations in complex natural microbial communities remain unclear. Here we investigated the development of a methanotrophic microbiome following subsurface mud eruptions at Håkon Mosby mud volcano (1250 m water depth). Freshly erupted muds hosted deep-subsurface communities that were dominated by *Bathyarchaeota*, *Atribacteria* and *Chloroflexi*. Methanotrophy was initially limited to a thin surface layer of *Methylococcales* populations consuming methane aerobically. With increasing distance to the eruptive center, anaerobic methanotrophic archaea, sulfate-reducing *Desulfobacterales* and thiotrophic *Beggiatoaceae* developed, and their respective metabolic capabilities dominated the biogeochemical functions of the community. Microbial richness, evenness, and cell numbers of the entire microbial community increased up to tenfold within a few years downstream of the mud flow from the eruptive center. The increasing diversity was accompanied by an up to fourfold increase in sequence abundance of relevant metabolic genes of the anaerobic methanotrophic and thiotrophic guilds. The communities fundamentally changed in their structure and functions as reflected in the metagenome turnover with distance from the eruptive center, and this was reflected in the biogeochemical zonation across the mud volcano caldera. The observed functional succession provides a framework for the response time and recovery of complex methanotrophic communities after disturbances of the deep-sea bed.

## Introduction

The ocean seabed is the largest methane reservoir on Earth, comprising this climate-relevant gas in the form of semi-stable methane hydrates, as gas bubbles or dissolved in porewater. Globally, most of the methane rising from deeper subsurface layers is oxidized by methanotrophic microbial communities before it can reach the hydrosphere [1]. The methanotrophic communities in the seabed are diverse, but dominated by relatively few globally distributed types [2]. The thin oxic surface layer of methane-rich sediments is often inhabited by aerobic methanotrophic bacteria of the *Methylococcales* [2–5]. Anoxic subsurface layers, where methane and sulfate overlap, are inhabited by consortia of anaerobic methanotrophic archaea (ANME) and their partner bacteria of the sulfate-reducing *Desulfobacterales* [6–9]. These methanotrophic communities, also referred to as the microbial methane filter, remove >90% of the methane in undisturbed continental margin sediments [1]. Methanotrophs also play an important role in methane removal at shallow [10, 11] and deep-sea gas-emitting seep habitats [12, 13]. Hence, only a small fraction of the seabed methane escapes from these sediments to the hydrosphere and atmosphere. However, the microbial methane filter at geologically highly dynamic seeps such as mud volcanoes has a lower efficiency, removing only 10−30% of the rising methane [14, 15]. Understanding the causes for these different efficiencies, as well as the time scales needed for the establishment of an efficient methane filter, is crucial in order to assess the consequences of natural and man-made seafloor disturbances, such as rapidly dissociating hydrates [16, 17], mud slides, eruptive mud volcanoes [14] or large oil spills [18–20].

Here we study the development of a deep-sea microbiome disturbed by seafloor mixing due to gas eruptions and mud slides at the actively gas-emitting Håkon Mosby Mud Volcano (HMMV) on the Norwegian continental slope. Marine mud volcanoes are seabed structures formed by upward migration of subsurface gasses together with fluids and sediments, from hundreds of meters to several kilometers depth by buoyancy and gravitational forces [21]. They are an important source of the greenhouse gas methane, globally emitting an estimated 27 Tg per year [22]. It has been speculated that the reduced efficiency of the microbial methane filter at mud volcanoes could be due to the low availability of electron acceptors, since the sediments are purged with anoxic subsurface fluids rising with the gas [14, 23]. Other factors may be fluctuating temperatures, or frequent disturbances by mud mixing, which affect the growth of methanotrophs [24]. To investigate this further we compared the biogeochemistry and microbial community composition between recently disturbed, partially recovered, and undisturbed seafloor, using time-series observations and sampling of the Håkon Mosby in the framework of the deep-sea observatory “LOOME—Long term observations of mud volcano eruptions” (2003−2010). The hypotheses tested were (1) that the subsurface microbial signature of freshly erupted muds disappears with exposure to deep oxygenated seawater, (2) that freshly erupted muds lack complex methanotrophic communities and hence may have a low capacity to remove methane, and (3) that it needs years to develop complex cold-seep communities due to the slow generation times and cold temperatures.

## Results and discussion

**Fig. 1:**
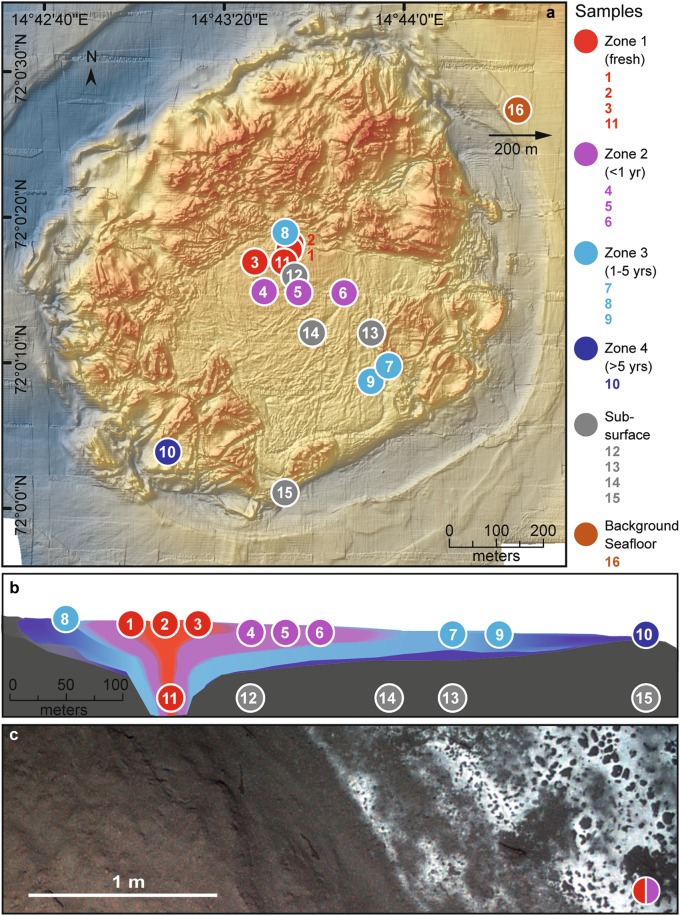
Bathymetry and seafloor imaging of Håkon Mosby mud volcano (HMMV). Samples originated from four zones, which differed in biogeochemistry and distance to the active center, i.e. time since the last eruption (**a**). Samples 1−10 and 16 are from surface muds (top 0−10 cm), samples 11−15 are from >2 m depth (**b**). Image of the sediment surface close to the center of HMMV. Freshly erupted muds flowing across consolidated muds that are covered with white mats of sulfur-oxidizing bacteria (**c**)

**Fig. 2:**
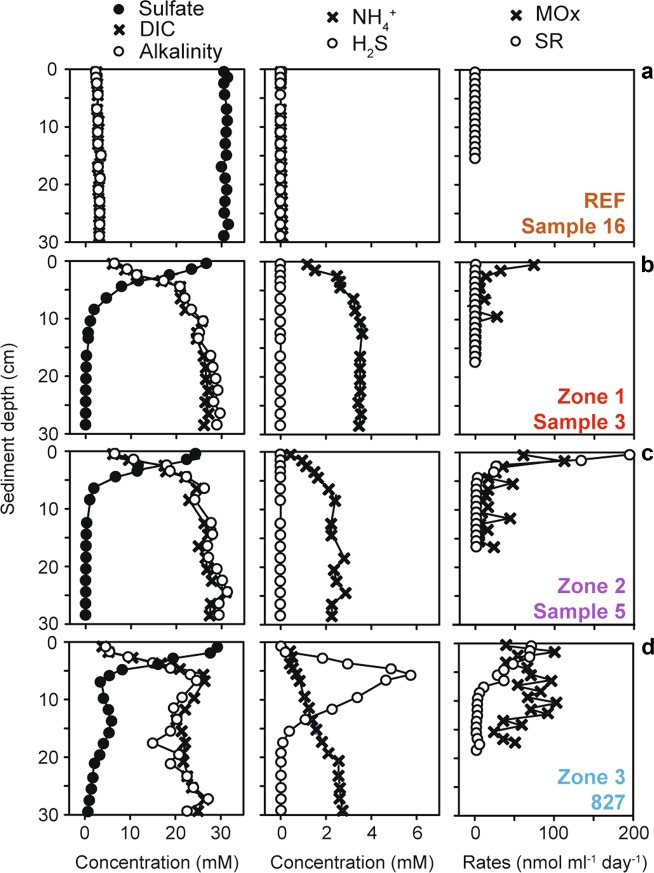
Biogeochemistry of surface sediments of HMMV and of a reference site outside of the mud volcano. In the reference sediment (**a**) outside of the HMMV caldera around 500 m upslope of the mud volcano, dissolved inorganic carbon (DIC), sulfate and alkalinity showed typical background concentrations, ammonium (NH_4_) and sulfide (H_2_S) were not detected, and there was no measurable methane oxidation (MOx) or sulfate reduction (SR). Sediments at the center of HMMV (**b**) show an upward transport of sulfate-depleted subsurface fluids enriched in DIC and NH_4_ and show low MOx rates. SR is first detectable in sediments of zone 2 (**c**) and H_2_S production is first detectable in aged sediments of zone 3 (**d**). Note: MUC827 is a parallel core of Sample 9 (MUC823) originating from the same dive and same area. All profiles were measured on the same expedition in 2010. For details as well as pore-water concentrations and rates of other samples in zones 1−4, see Table 2 and ref. [61]

We investigated the seabed microbial community in mud flows of the HMMV (72°N, 14°44′E, 1250 m water depth) during research campaigns in August 2009 and September 2010. In this period, the long-term geophysical recordings of the LOOME observatory (Fig. 1, S1; [25]) measured three major and 12 minor eruptions that occurred every 3−4 weeks. From this eruption pattern, detected by our deployed instruments and visual observations, we were able to infer an average mud flow velocity of 0.4 m day^−1^ across the HMMV center [25]. The mud velocity was used to convert sample distance to the eruptive central conduit into time. This space-for-time substitution approach used in ecological analyses of disturbances [26] allowed us to infer the spatiotemporal development of the methanotrophic microbiome. After visual seafloor inspection by ROV Quest and AUV Sentry, and biogeochemical sampling, we categorized the center muds into four zones (Figs. 1 and 2, S1; [27]). Zone 1 covered an area of about 50 × 90 m at the HMMV center. It consisted of fresh subsurface muds that were deposited during several gas eruptions recorded in 2009−2010 (Fig. 1c, Fig. S2; [25]). Zone 2 consisted of older subsurface mud flows southeast of the active center. The muds had a smooth, slightly rippled surface and were exposed to seafloor conditions for 1−2 years according to the morphology of the seabed and the measured flow velocity of the muds. Zone 3 were muds > 200 m away from the eruptive center, with thin thiotrophic mats, exposed to cold bottom waters already for 2−5 years. As a fourth zone we sampled the hummocky rim around the active center of HMMV. These sediments are stabilized by layers of hydrate at a few meters sediment depth [28]. They are not physically mixed by the center eruptions, as evident by comparative high-resolution mapping of the structure in 1996 [29, 30], and by the dense coverage of long-lived siboglinid tubeworms (Fig. S2F), which were absent in zones 1–3.

### Biogeochemistry and microbial methanotrophic rates change with time and distance from the eruptive center

The fresh warm subsurface muds exposed during eruptions were saturated with methane as indicated by spontaneous in situ degassing, and had high concentrations of dissolved inorganic carbon (DIC), alkalinity and ammonium, indicative of a deep-subsurface origin of the pore fluid (Table 2, Fig. 2b, c). The fluids originate from a depth of up to 3 km below the seafloor, where the central conduit of HMMV is rooted [28, 31]. The methane carbon isotopic signature of the dissolved gas in the warm sediments is similar to that of the surrounding gas hydrates with around δ^13^C = −60‰ (PDB), indicating a mixed thermogenic/biogenic origin in the deep subsurface [32]. Due to holes and cracks from degassing of the center muds (Fig. S2A), sulfate-containing bottom water percolated 5−10 cm into the seafloor. In the exposed surface muds 9 months after the main eruption, we measured methane oxidation (MOx—total aerobic and anaerobic methane oxidation) rates of up to 80 nmol ml^−1^ day^−1^. These are low rates for seep ecosystems [6], especially when considering the high availability of methane and oxygen at the surface, or methane and sulfate in HMMV subsurface sediments. Sulfate reduction (SR) was not detectable, and the concave shape of the sulfate gradient (Fig. 2b) is explained by the downward diffusion of sulfate against an upward flux of subsurface fluids [33]. In zone 2, which still showed ripples from the mud eruptions (Fig. S2D), MOx rates were higher than in zone 1, peaking at 100 nmol ml^−1^ day^−1^ at the surface (Fig. 2c); SR rates peaked in the top few centimeters, but still no free sulfide was detected in the porewaters. Zone 3 is characterized by mats of sulfide-oxidizing bacteria (Figs. S1B, C, S2D, E). Here, the sulfate profile showed substantial consumption in the upper centimeters, and sulfide concentration reached almost 6 mmol l^−1^ (Fig. 2d). Integrated SR rates of zone 3 sediments measured during six expeditions between 2001 and 2010 (Table 2) consistently showed the high rates that are typical for a well-established community of anaerobic methanotrophs (18 ± 4 mmol m^−2^ d^−1^; mean ± S.E.; *n* = 18; Table 2; [34]). These rates are around 60-fold higher than average sulfate reduction rates of nonseep impacted shelf sediments [35]. Sulfide production peaked in surface sediments of zone 3 (1.19 ± 0.15 mmol l^−l^; *n* = 110), whereas sulfide concentrations were low in zones 1 and 2 (Table 2) confirming that AOM was not established in fresh sediments. Porewater analyses as well as methane oxidation and sulfate reduction rate measurements performed on these six expeditions support the lateral zonation across the caldera. This zonation is in accordance with the geophysical model, in which centrally rising mud and gas are laterally transported and eventually sink in upon degassing [25]. Our biogeochemical results strongly suggest that over the period of at least one decade AOM continuously established in muds of the same distance to the eruptive center—i.e. muds of a similar age—despite a continuous mud flow.

### Subsurface communities get transported to surface sediments due to mud volcanism

**Fig. 3:**
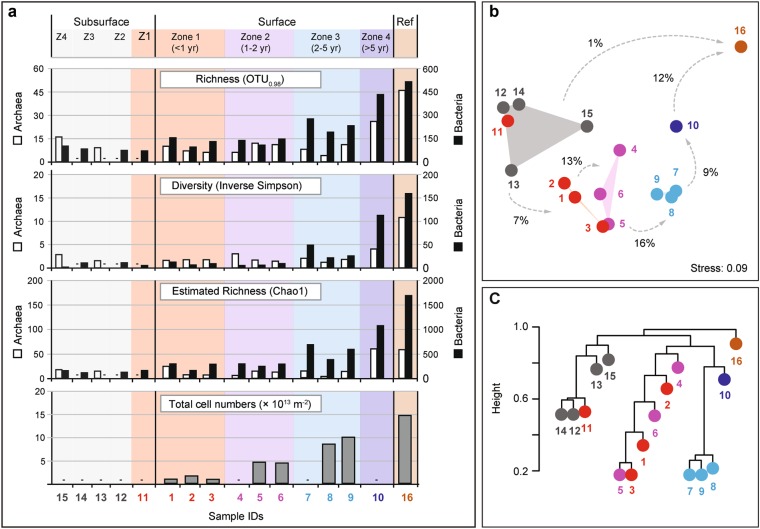
Alpha and beta diversity, and total cell abundance across HMMV sediments. **a** Archaeal and bacterial diversity was determined using OTUs (Operational taxonomic units at 98% 16S rRNA V4-V6 gene sequence identity, corresponding to the recommended taxonomic threshold for microbial species [74]). OTU data were used to assess observed richness, Inverse Simpson diversity, and Chao1 estimated richness. Note: The axes for archaeal and bacterial values differ by one order of magnitude. Total cell numbers were determined by Acridine Orange Direct Counts and were integrated over the top 10 cm sediment depth. Dashes denote missing data points. **b** Shift of microbial community structure in HMMV sediments as visualized by nonmetric multidimensional scaling (NMDS) using relative sequence abundance of archaeal and bacterial OTUs. Color indicates the sample origin (Subsurface = gray polygon; Zone 1 = red; Zone 2 = purple; Zone 3 = light blue; Zone 4 = dark blue; Reference site = brown). The percentages of microbial OTUs that are shared between zones (numbers next to arrows) are based on presence−absence data, i.e. showing that only 1% of OTUs that are present in the subsurface are also found in the surface sediment of the reference site. The microbial communities of the subsurface and of zones 1−3 were all significantly different from each other (ANOSIM based on presence/absence data: *R* = 0.7, *p* = 0.001). Zone 4 and the reference site could not be included in the ANOSIM as there was only one sample retrieved. **c** Dendrogram showing hierarchical clustering of the samples based on archaeal and bacterial OTUs.

**Fig. 4:**
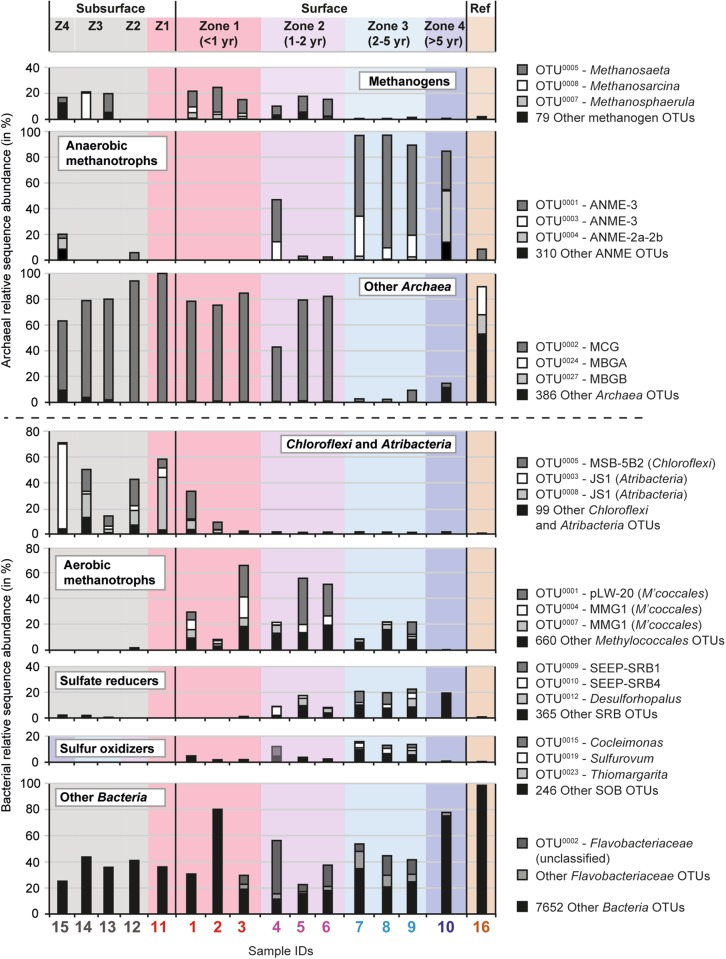
Succession of microbial clades in sediments of HMMV based on relative abundance of OTUs. Together the bars of one sample add up to 100% archaeal and 100% bacterial relative sequence abundance focusing on functionally relevant clades involved in the methane and sulfur cycle. Each bar in these panels shows the top 2−3 most sequence abundant OTUs and all remaining OTUs (Other) that belong to this subset. A complete table with archaeal and bacterial OTUs, as well as a summary table of relative abundances of key populations (e.g. ANME-3) is available at PANGAEA, see ref. [61]

We analyzed archived subsurface samples from the pre-study phase in 2003 and additionally assessed the microbial community composition of ten surface (S) and five subsurface (D) sediment samples across the same zones sampled after the eruptions. The microbial communities in the warm (10−20 °C) subsurface sediments (3.8−2.5 m below seafloor) of the HMMV caldera were characterized by a relatively low archaeal and bacterial alpha diversity, and showed a low community turnover, i.e. replacement of microbial taxa across zones (Figs. 3, 4, Fig. S3). These simple and homogeneous communities support the geophysical model of a uniform, warm subsurface mud layer filling the central chimney of HMMV [25, 28, 36]. Interestingly, the subsurface communities were most similar to the surface communities of zone 1 (Fig. 3b, c; Table S2), which is in accordance with the observed upward transport and deposition of the subsurface sediments by gas eruptions (Fig. S2A). Subsurface and also surface sediments of Zone 1 contained typical heterotrophic deep biosphere clades, such as *Bathyarchaeota* (Miscellaneous Crenarchaeotic Group), *Chloroflexi* and *Atribacteria* (candidate division JS1) [37, 38] as well as *Peptococcaceae* and methanogenic *Methanosaeta* (Fig. 4, Fig. S4). The latter four clades were suggested to form a syntrophic network, degrading proteins and fatty acids under methanogenic conditions [39]. *Bathyarchaeota*, which comprise organisms that also degrade detrital proteins [40], greatly dominated the archaeal community in the freshly deposited muds (Fig. 4, S4). We detected many genes involved in fermentation and methanogenesis (Fig. S4, S5A) in the subsurface metagenomes, while genes for sulfate reduction, sulfur oxidation or methane oxidation were absent or very rare. The subsurface communities in the central mud conduct of HMMV may be fueled by organic compounds transported with rising porewater fluids from the deep subsurface. The subsurface and surface community of zone 1 contained very few ANME-3 sequences (<1% relative sequence abundance), and few genes affiliated with *Methylococcales*, sulfate-reducing bacteria (SRB) and sulfur-oxidizing bacteria (SOB) (Fig. S4), in accordance with the biogeochemical profiles of the freshly exposed muds (Fig. 2, Table 2).

**Fig. 5:**
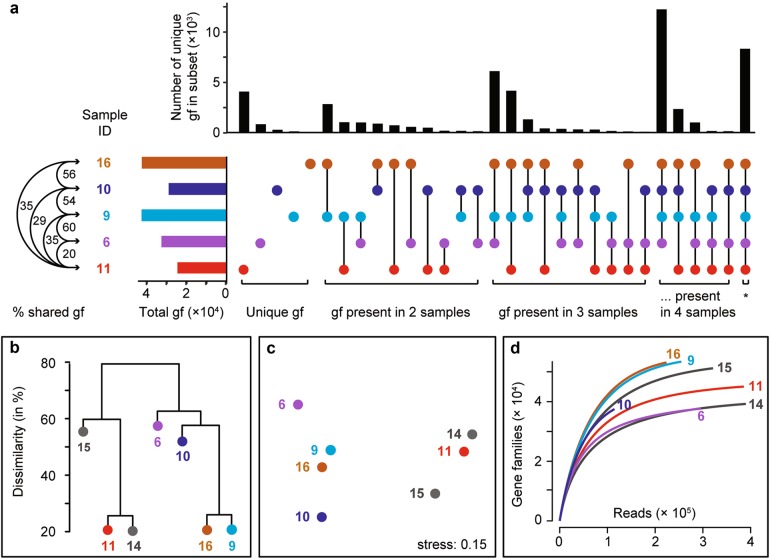
Richness and turnover of gene families (gf) across HMMV sediments. **a** The “UpSet” diagram is analogous to a Venn diagram and is based on a presence/absence matrix. The vertical bars represent the number of gene families that were exclusively found in a respective combination of metagenomes. The total number of gene families found in a metagenome is shown as horizontal bar, percentages of gene families shared between metagenomes are indicated. Note: For clarity the subsurface samples 14 and 15 were omitted in the UpSet diagram, without changing the overall trends. Gene family richness and percent shared gene families of all seven metagenomes are shown in Tables S5 and S6, respectively. Turnover of gene families between metagenomes is visualized by dendrogram (**b**) and nonmetric multidimensional scaling ordination (**c**). **d** Rarefaction indicates that most gene families, and possibly metabolisms, that are present at HMMV were detected

By comparative sequence analysis, we tested if the subsurface clades deposited at the surface, such as *Atribacteria* [41], would persist in the surface muds with increasing exposure and distance from the central mud conduit. The subsurface-derived clades decreased in relative sequence abundance with increasing distance to the center. In zone 3, these clades were barely detectable in the sediments. The subsurface microbial signature was replaced by that of typical seep communities within around 2 years. Only 3−7% of the operational taxonomic units (OTUs; at 98% 16S rRNA gene V4-V6 sequence identity) were shared between subsurface and surface muds (Fig. 3, Table S2) and even less (1% shared OTUs) with the reference site that was characterized by typical oligotrophic deep-sea sediment organisms, such as *Xanthomonadales* and *Thaumarchaeota* [42]. This diversity pattern was also confirmed based on the turnover of gene families in the community metagenomes (Fig. 5a). Only 18−35% of the gene families found in the subsurface metagenome of zone 1 (sample 11) were found in the surface metagenomes, while 66 and 77% gene families were shared between sample 11 and the other two subsurface metagenomes (Table S6). Similarly, the surface metagenomes shared between 52 and 79% gene families among each other (60 ± 9.8 %, mean ± SD, *n* = 6), but only 18−48 % with the subsurface metagenomes (33 ± 9.4%, mean ± SD, *n* = 12). This suggests that subsurface-derived genes, and hence subsurface community functions, were rapidly depleted in the surface muds. Subsurface microbes have longer generation times compared to surface bacteria [43–45]. In addition, they were likely repressed by the exposure to the seafloor conditions; i.e. cold temperatures of −1 °C and high oxygen concentrations (>280 μM; [46]), and were eventually overgrown by others.

### Surface communities developing with distance from the eruptive center

**Fig. 6:**
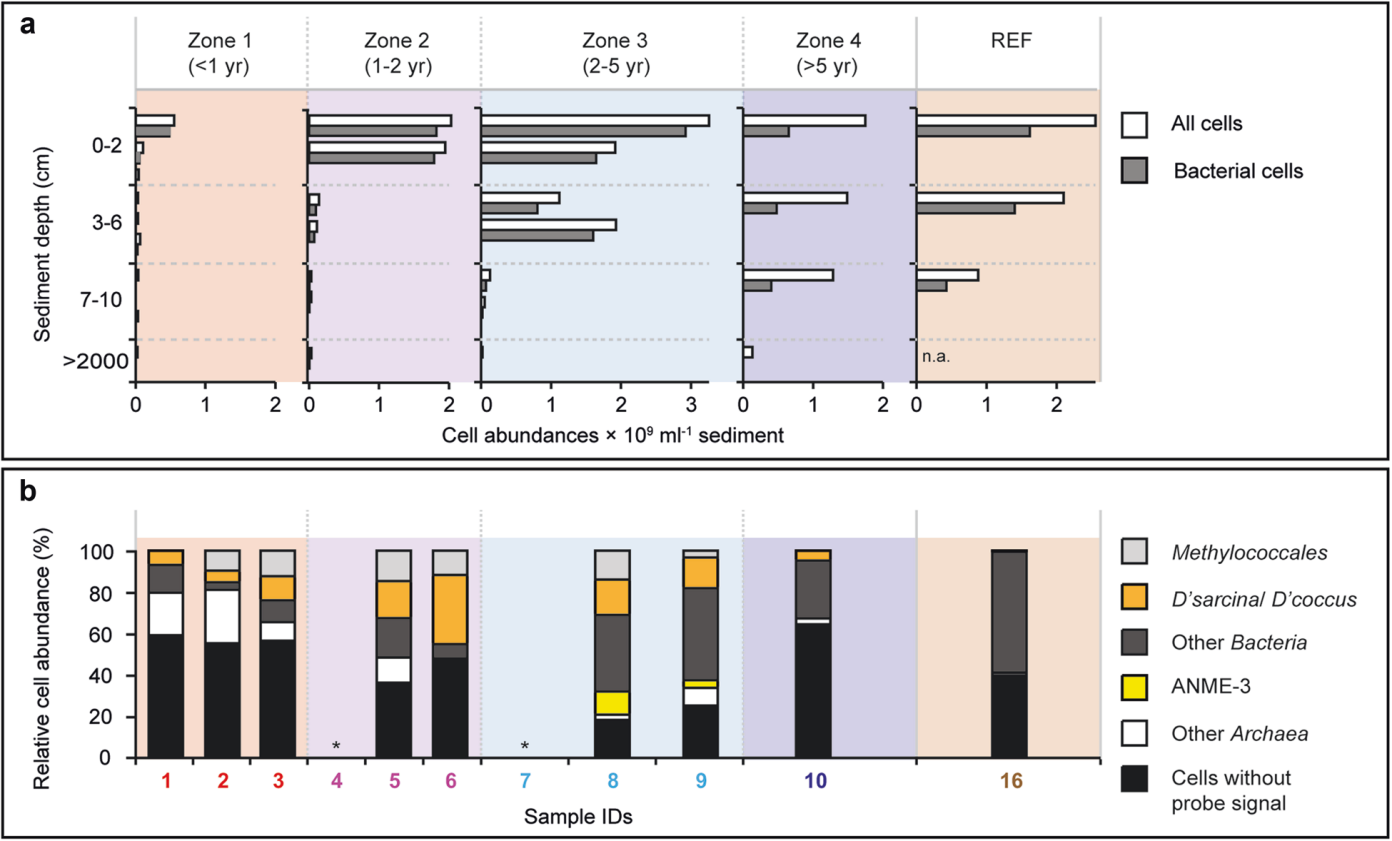
Total and relative cell abundance of microbial clades in top sediment layers of HMMV. Total microbial cell numbers (**a**) were assessed with DAPI (white bars) and compared to bacterial cell abundance (probe: EUB338-I-III, gray bars). Replicate samples were available for zones 1−3 (zone 1: *n* = 3, zone 2/3: *n* = 2), shown as multiple bar pairs. **b** Relative cell abundances based on single-cell counts using CARD-FISH with specific probes for *Bacteria* (probe EUB338 I-III), *Methylococcales* (probe MTMC-701 and competitor probes), *Desulfosarcina/Desulfococcus* (probe DSS658), *Archaea* (probe Arch915), and ANME-3 (probe ANME3-1249 and helper probes). *Bacteria* that did not overlap with *Methylococcales* or DSS are denoted “other *Bacteria*”*. Archaea* that did not overlap with ANME-3 are referred to as “other *Archaea*”. “Cells without probe signal” were only stained by the nucleic acid stain DAPI and not by general archaeal or bacterial probes. Relative abundances were averaged over the top ten centimeters; all three layers are shown in Figure S7. Note: The relative cell abundances for ANME-3, *Desulfosarcina/Desulfococcus* and *Methylococcales* are underestimated as these clades formed cell aggregates that were not included in the single-cell counts. Probe details are given in Table S3; detailed values and cell counts are archived [61]

Using the mud flow velocities measured by LOOME observatory and space-by-time substitution, we investigated how the microbiome composition and metabolic capabilities of the surface communities would shift across the different zones with time, and if these changes were in accordance with the changes in biogeochemical rates (Fig. 2; Table 2). The analysis of 16S rRNA gene amplicons confirmed that community structure was significantly different between the zones, as tested by ANOSIM (*R*_Arch_ = 0.7, *p*_Arch_ < 0.01; *R*_Bac_ = 0.5, *p*_Bac_ < 0.01, Fig. S3). Also, the communities were more similar between adjacent zones than between zones that were further apart, e.g. zone 1 shared more OTUs with zone 2 than with zone 3 (Table S2). Total cell counts increased with distance to the active center (Figs. 3, 6a). Relative cell abundances of key microbial clades involved in methanotrophy and sulfate reduction also changed between the zones (Fig. 6b), increasing twofold from zone 1 to 2. Further, we observed a strong increase in microbial richness and evenness in zone 3 compared to surface sediments of zone 1 and 2 (Fig. 3). Microbial diversity continued to increase in the zone 4 sample and peaked in the reference site. Around 85% of archaeal and bacterial OTUs of the freshly erupted muds of zone 1 were replaced within 1−2 years of exposure to surface conditions (Fig. 3, Table S2). Yet, the communities of zones 1 and 2 still shared key organisms (e.g. *Methylococcales* and *Desulfobacterales*), especially those samples taken at a similar distance to the central conduit (e.g. Samples 3 and 5, Fig. 3). Zone 3 sediments reached total cell numbers of 3 × 10^9^ cells ml^−1^, similar to the community density in the stable hummocky rim (zone 4). Zone 4 harbored the most complex microbial communities at HMMV, with the highest diversity and evenness (Fig. 3). The overall community development observed at the level of 16S ribosomal RNA gene diversity and turnover was also confirmed by the diversity and turnover of metabolic gene families (Fig. 5; Figs. S4, S5). Rarefaction curves showed that the expected number of gene families was highest in the consolidated muds of zone 3 and the reference site (Fig. 5d) and generally lower in freshly exposed sediments and subsurface muds. Similarly, the total number of observed gene families was highest in the metagenome of surface sediment from the reference site and low in the sediments of the active center. At the same time the number of unique gene families that exclusively occurred in one metagenome was high in the active center and lowest in the reference site (Fig. 5a). This indicates that with distance from the active center the surface sediments are becoming increasingly diverse, but also increasingly similar on the level of community functions.

### Development of aerobic methanotrophic populations

In zones 1 and 2, OTUs affiliated with aerobic methanotrophic *Methylococcales* were abundant in the top cm of surface sediments exposed to the oxygen-rich cold bottom waters, as shown by relative sequence and cell abundances (Figs. 4, 6) and a high number of 16S rRNA and *pmoA* gene sequences in the metagenome (Fig. S4, S5B). The *Methylococcales* reached 5 × 10^8^ cells ml^−1^ sediment (3.1 ± 1.9 × 10^8^ cells ml^−1^ sediment, mean ± SD, *n* = 6; Table 2) already after a few months of exposure of the subsurface muds (Fig. S6). This peak in cell abundance of aerobic methylotrophs in fresh muds was observed in previous expeditions [14, 15, 47], suggesting that these organisms rapidly colonize fresh HMMV muds in general. In the thin sediment surface layer where oxygen is available, *Methylococcales* can respond faster to the high supply of methane, as their higher energy yield supports faster growth rates compared to those of anaerobic methanotrophs [48–51]. Also, aerobic methylotrophs could relatively rapidly colonize freshly exposed gassy muds, as they can disperse with bottom waters [2, 52]. Assuming that representatives of this group would settle on the freshly deposited muds by sediment resuspension across the mud volcano, and applying a mud transport rate of 0.4 m per day between sediments of zones 1 and 2 as observed in this time period [25], the aerobic methylotrophs could have achieved a net growth rate of 0.01 day^−1^ corresponding to a doubling time of 60 days. This rate is similar to that calculated for *Methylococcales* in arctic, boreal swamps (0.02 day^−1^; [53]). In comparison, cold-water anaerobic methanotrophs commonly show growth rates of around (0.003 day^−1^) [48]. This finding supports previous hypotheses, that aerobic methanotrophs dominate surface sediments of emerging methane leaks and young seep systems [24, 54, 55]. However, spatially, they can only occupy a small niche due to limited oxygen penetration into the seafloor [12, 15, 54].

### Development of anaerobic methanotrophic communities and sulfur-oxidizing bacteria

Cell counts and sequences showed that anaerobic methane-oxidizing archaea (ANME) and their sulfate-reducing partner bacteria were rare in the freshly exposed center sediments of zone 1 (Figs. 4, 6; Fig. S7). Free-living sulfate-reducing bacteria (SRB) increased tenfold in relative abundance from zone 1 to zone 2, the latter harboring 2.4 ± 1.5 × 10^8^ SRB cells ml^−1^ sediment (Table 2). Zone 3 had similar SRB counts as zone 2, but in addition harbored large numbers of ANME/SRB consortia (Fig. 6, Table 2). The sulfide that was produced by these AOM consortia (Fig. 2d, Table 2) supported the growth and establishment of sulfur-oxidizing bacteria (SOB), forming white mats covering zone 3 (Fig. S1, S2D, E). In comparison, the thiotrophs were rare in sequence abundance in sediments of zone 1. Their relative sequence abundance increased between zones 2 and 3, where an increasing amount of oxidative *dsrAB* genes were detected in the metagenomes (Figs. S4, S8). The archaeal community of zone 3 was dominated by a single ANME-3 OTU. This OTU had a relative sequence abundance of below 0.1% in freshly exposed subsurface muds of zone 1, 2−30% in zone 2 and comprised 63−88% of all archaeal sequences in zone 3, indicating a significant increase of the population with time and distance from the mud and gas conduit, but also suggesting a slow doubling time of 100−200 days. This estimated doubling time of ANME-3 in situ corresponds to a growth rate of 0.003−0.006 day^−1^ which is in good agreement to the rates estimated by in vitro experiments with psychrophilic ANME-2 (0.003 day^−1^; [48]). All populations of ANME, SRB and SOB (Figs. 4, 6) and their metagenomic signatures, especially the relative abundances of *mcrA* and *dsrAB* genes (Figs. S4, S5A, S8) showed the same pattern. They were rare in subsurface and freshly exposed muds, and became abundant in surface sediments with increasing distance from the center and thus exposure time of the muds. This demonstrates that slow-growing, and initially rare hydrocarbon-consuming microorganisms are able to out-compete others at cold seeps when the conditions are favorable and the time-scale permits [56, 57]. In the undisturbed zone 4, ANME-2a and ANME-3 archaea had similar relative sequence abundances (Fig. 4), indicating that these consolidated sediments harbor niches for more ANME clades, as compared to the disturbed sediments of the caldera, which were highly dominated by ANME-3. In zone 4, very little methane reaches the surface sediment due to active methanotrophic communities at the roots of the tubeworms in ~60 cm depth [47], resulting in very low methane oxidation rates at the surface (Table 2). The low temperature and stability of the sediments as well as the presence of hydrates below 0.5−1 m sediment depth may explain why the investigated surface sediment are of low activity and share 12% OTUs with surface sediment of the nonmethane reference site (Fig. 3, Fig. S3). Together, the observation of community succession on mud flows at HMMV match previous experiments with wood and whale falls showing that deep-sea methanogenic, methanotrophic and thiotrophic clades need years to develop functional communities on allochthonous surfaces and energy supplies [58–60].

## Conclusion

Here we studied the development of a deep-sea methanotrophic microbiome on mud flows of a methane-emitting mud volcano in situ. We were able to sample the communities of freshly exposed subsurface muds, and compare them to their source community, as well as to increasingly developed seep and nonseep communities outside the caldera. Changes in biogeochemical rates with increasing distance to the eruptive center of the mud volcano were mirrored by changes in the corresponding metabolic genes and cell counts of the respective clades. At the level of the whole microbial metagenome, both 16S rRNA sequence turnover as well as the diversity of metabolic gene families showed a pattern of increasing complexity with increasing development of the methanotrophic assemblages, supporting a rich and diverse bacterial and archaeal community. We were able to confirm our initial hypothesis based on biogeochemical measurements that subsurface communities of bacteria and archaea are replaced by pioneering aerobic methanotrophs and later complex anaerobic methanotrophic and thiotrophic communities. Even when electron donors and acceptors are not limiting, the succession of benthic deep-sea bacterial and archaeal populations may need years, before the typically high diversity and evenness of deep-sea sediment communities is reached. Our findings indicate that loss of seafloor integrity—in this case by gas eruptions and mud mixing—and thereby the local decline of active and complex methanotrophic communities can explain the low efficiency of methane consumption that is globally observed at active mud volcanoes. Over several years, a seep microbiome can develop from initially rare populations to a complex community, in this case study evidenced by increasing cell and sequence numbers, and increasing diversity, of aerobic and anaerobic methanotrophs and thiotrophs. The observed functional succession provides insights into the response time and recovery of complex microbial communities to natural and anthropogenic disturbances in the deep sea.

## Materials and methods

### Sampling sites

Surface sediment samples (0−10 cm) at HMMV are exposed to the cold Arctic bottom water, and generally have an ambient temperature of −1 °C. They were recovered either by TV-guided Multicorer or by push cores using the remotely operated vehicle Quest (Marum, University Bremen) (Table 1). Subsurface sediments of all zones (>2 m below sea floor) were obtained by gravity corer. Their temperature at 4 m below the seafloor ranged from around 15 °C in the center to around 3 °C at the hummocky rim [36]. After recovery, sediments were immediately subsampled in a refrigerated container (0 °C) and further processed for biogeochemical analyses or preserved at −20 °C for later DNA analyses. Further details to the geographic locations, dates of sampling, and all contextual data are provided in the supporting information Table 1 and in the public archive for Earth and environmental data PANGAEA; see ref. [61].

**Table 1:** Sample overview

Sample ID	Sample zone	Sediment depth (mbsf)	VAMPS project label^a^	PANGAEA event label—relative cell abundances^b^	PANGAEA event label—porewater chemistry and biogeochemistry^b^	Date (MM/DD/YYYY)	Latitude	Longitude
**1**	**Zone 1**	0-0.1	RAM_Av6v4_PS74_2_169_1_PUC3	PS74/169-1_PUC-1	PS74/169-1_PUC-15	7/23/2009	72.00500	14.72640
**2**	**Zone 1**	0-0.1	RAM_Av6v4_PS74_2_168_1_MUC	PS74/168-1	PS74/168-1	7/23/2009	72.00470	14.72420
**3**	**Zone 1**	0-0.1	RAM_Av6v4_MSM16_2_838_1_MUC	MSM16/2_838-1	MSM16/2_838-1	10/1/2010	72.00480	14.72615
**4**	**Zone 2**	0-0.1	RAM_Av6v4_PS64_312_1_MUC	PS64/312-1	PS64/312-1	6/28/2003	72.00420	14.72490
**5**	**Zone 2**	0-0.1	RAM_Av6v4_MSM16_2_847_1_MUC	MSM16/2_847-1	MSM16/2_847-1	10/2/2010	72.00417	14.72702
**6**	**Zone 2**	0-0.1	RAM_Av6v4_MSM16_2_855_1_MUC	MSM16/2_855-1	MSM16/2_855-1	10/4/2010	72.00403	14.72980
**7**	**Zone 3**	0-0.1	RAM_Av6v4_PS64_317_PUC_17	PS64/317_PUC-17	PS64/317_PUC-17	6/30/2003	72.00260	14.73145
**8**	**Zone 3**	0-0.1	RAM_Av6v4_PS74_2_172_1_PUC131	PS74/172-1_PUC-131	PS74/172-1_PUC-136	7/25/2009	72.00520	14.72623
**9**	**Zone 3**	0-0.1	RAM_Av6v4_MSM16_2_823_1_MUC	MSM16/2_823-1	MSM16/2_826-1	9/29/2010	72.00311	14.73134
**10**	**Zone 4**	0-0.1	RAM_Av6v4_PS64_326_PUC_12	PS64/326_PUC-12	PS64/326_PUC-12	7/2/2003	72.00098	14.70217
**11**	**Zone 1**	3.8	RAM_Av6v4_PS64_332_1_GC	PS64/332-1	PS64/332-1	7/2/2003	72.00470	14.72620
**12**	**Zone 2**	2.5	RAM_Av6v4_PS64_372_1_GC	PS64/372-1	PS64/372-1	7/11/2003	72.00440	14.72660
**13**	**Zone 3**	4.6	RAM_Av6v4_PS64_371_1_GC	PS64/371-1	PS64/371-1	7/11/2003	72.00330	14.73130
**14**	**Zone 3**	2.8	RAM_Av6v4_PS64_373_1_GC	PS64/373-1	PS64/373-1	7/11/2003	72.00340	14.72770
**15**	**Zone 4**	3.8	RAM_Av6v4_PS64_336_1_GC	PS64/336-1	PS64/336-1	7/5/2003	72.00030	14.73550
**16**	**REF**	0-0.1	RAM_Av6v4_MSM16_2_809_1_MUC	MSM16/2_809-1	MSM16/2_809-1	9/26/2010	72.00666	14.74766

*mbsf* meters below seafloor

^a^This label denotes archaeal datasets. Corresponding bacterial datasets can be accessed under (e.g. RAM_Bv6v4_*).

Sequencing raw data and metadata can be accessed under: https://vamps.mbl.edu/

^b^Contextual data can be accessed under doi: 10.1594/PANGAEA.861266 (https://doi.pangaea.de/10.1594/PANGAEA.861266)

### Biogeochemistry

Porewater and turnover rates were measured in surface sediment cores obtained in 2010 using methods described previously [62]. We show four profiles in detail (Fig. 2, MUC-809, MUC-827, MUC-838, MUC-847), all other measurements from 2010 are included in Fig. S8 and the summary Table 2 and can be accessed from the data publisher PANGAEA; see ref. [61]. Biogeochemical parameters of sediments from 2003 and 2009 have been reported previously [14, 15]. The pore water was extracted with Rhizons in 1 cm resolution and immediately fixed in 5% zinc acetate (ZnAc) solution for sulfate, and sulfide analyses. The total sulfide concentrations (H_2_S + HS^−^ + S^2−^) were determined using the diamine complexation method [63]. DIC and alkalinity were measured using the flow injection method (detector VWR scientific model 1054) [64]. Nutrients were determined with a Skalar Continuous-Flow Analyzer [65]. Sulfate reduction (SR) and anaerobic oxidation of methane (AOM) were measured ex situ by the whole core injection method [66] as previously described [67, 68]. Refer to SI for details on biogeochemical analyses. All cores except the reference site were degassing methane after retrieval; hence we could not measure true in situ methane concentration in pore waters. It was previously estimated that the in situ concentrations of methane in the gassy HMMV center could reach 100 mM [23].

**Table 2:** Porewater chemistry, cell counts, methane oxidation, and sulfate reduction rates^a^

	SO_4_^2−^ (mM)	H_2_S (mM)	DIC (mM)	Alkalinity (mM)	SiO_4_^4−^ (µM)	PO_4_^3−^ (µM)	NH_4_^+^ (µM)	NO_3_^−^ + NO_2_^−^ (µM)	NO_2_^−^ (µM)	Mox Rate [mmol m^2^ d^-1^]	SR Rate [mmol m^2^ d^-1^]	Cell counts probe DSS-658^b^	Cell counts probe MTMC-701^b^	Cell counts probe ANME-1249^b^
**Zone 1**														
**Mean**	10.4	0.03	5.9	26.3	278	3.4	2379	*n.d*.	*n.d*.	3.3	0.7	0.3	0.2	0
**Standard Error**	1.6	<0.01	1.5	3.4	25.7	0.5	166	*n.d*.	*n.d*.	1.0	0.5	0.2	0.1	0
**No. Samples**	28	28	28	28	28	28	28	*n.d*.	*n.d*.	8	8	9	9	9
**Zone 2**														
**Mean**	13.1	0.001	10.1	15.5	237	2.1	1471	1.6	0.3	*n.d*.	*n.d*.	2.4	3.1	0
**Standard Error**	1.3	< 0.001	1.7	1.6	25.2	0.3	176	0.5	0.2	*n.d*.	*n.d*.	1.5	1.9	0
**No. Samples**	33	24	25	16	16	16	16	16	16	*n.d*.	*n.d*.	6	6	6
**Zone 3**														
**Mean**	11.0	1.19	3.8	18.8	225	31.4	963	*n.d*.	0.4	7.9	18.1	2.4	2.4	1.7
**Standard Error**	0.7	0.15	0.9	1.5	18.7	3.6	101	*n.d*.	0.1	1.4	4.1	0.9	1.7	0.6
**No. Samples**	121	110	88	26	25	25	25	*n.d*.	16	15	18	6	6	6
**Zone 4**														
**Mean**	30.2	0.47	5.6	0.5	23	3.9	140	6.1	0.6	0.9	*n.d*.	0.9	0.03	0.01
**Standard Error**	1.4	0.21	0.8	0.1	1.2	0.3	26	1.5	0.1	0.8	*n.d*.	0.3	0.02	0.01
**No. Samples**	23	10	11	11	11	11	11	7	11	4	*n.d*.	6	6	6
REF														
**Mean**	31.0	0	2.6	2.6	47	3.0	3	10.0	0.2	*n.d*.	*n.d*.	0.1	0.01	0
**Standard Error**	0.1	0	0.1	0.1	14.0	0.6	0.6	3.9	0.0	*n.d*.	*n.d*.	*n.c*.	*n.c*.	*n.c*.
**No. Samples**	7	7	7	7	7	7	6	7	7	*n.d*.	*n.d*.	3	3	3

*n.d.* not determined, *n.c.* not calculated, due to low number of samples

^a^Concentrations and cell counts have been averaged over the top 10 cm of sediment, and rates have been integrated over the top 10 cm of sediment using samples of respective zones from six expeditions: L’Atalante (2001), PS64 (2003), VKGD276 (2006), PS70 (2007), PS74 (2009), MSM16 (2010)

^b^Cell counts are given as cells ×10^8^ ml^−^^1^ sediment

### 16S rRNA gene V4-V6 amplicon pyrosequencing

DNA extraction was done in duplicates using 1 g sediment each and a commercially available extraction kit (UltraClean Soil DNA Isolation Kit, MoBio, Carlsbad, CA). The DNA was pooled and the V4-V6 hypervariable regions of archaeal and bacterial SSU rRNA genes were amplified using degenerate primers. The bacterial primers 1064R and 518F, or archaeal primers 517F and 1048R (for details see SI) were fused to Roche GSFLX amplicon sequencing adapters including 5 nt multiplexing barcodes. We generated PCR amplicons in triplicate 33 µl reaction volumes containing 1.0 U Platinum Taq Hi-Fidelity Polymerase (Invitrogen, Carlsbad, CA), 1× Hi-Fidelity buffer, 200 µM dNTP PurePeak DNA polymerase mix (Pierce Nucleic Acid Technologies, Milwaukee, WI), 1.5 mM MgSO_4_ and 0.2 µM of each primer. We added approximately 10−25 ng template DNA to each PCR and ran a no-template control for each primer pair. Amplification conditions were: initial denaturation 94 °C for 3 min; 30 cycles of 94 °C for 30 s, 60 °C for 60 s, and 72 °C for 90 s; final extension at 72 °C for 10 min. We assessed the quality, size and concentration of PCR products on a Perkin Elmer Caliper GX. Reads were demultiplexed and barcodes removed for submission. Sequence reads were submitted to a rigorous quality control procedure using mothur v30 [69] and a routine [2, 70] that included denoising of the flow grams [71], single-linkage preclustering [72] and the removal of chimeras [73]. Sequences were clustered at 98% ribosomal RNA gene V4-V6 sequence identity—corresponding to the recommended taxonomic threshold for microbial species [74]—and were taxonomically assigned using the SILVA taxonomy (SSURef v119, 07-2014 [75]). Further information about sequencing datasets and contextual data are available at PANGAEA [61].

### Analyses of V4-V6 amplicon data

Relative abundance of archaeal and bacterial OTUs (operational taxonomic units clustered at 98% sequence identity) is based on the original *mothur* output (Table S1). To calculate Inverse Simpson diversity indices and Chao1 Richness [76] the OTU abundance tables were rarefied to account for unequal sampling effort using 300 (Archaea) and 1000 (Bacteria) randomly chosen sequences without replacement using *mothur*. Bray−Curtis dissimilarities [77] between all samples were calculated and used for two-dimensional nonmetric multidimensional scaling (NMDS) ordinations with 20 random starts [78]. All analyses were carried out with the R statistical environment and the packages *vegan* [79], *labdsv* [80], as well as with custom R scripts (for details see SI).

### Shotgun metagenomics

DNA extraction using 3 g sediment (pooled from 0 to 10 cm sediment depth) was performed manually as previously described [81]. DNA was sheared using a Covaris and libraries were constructed with the Nugen Ovation Ultralow Library protocol and were amplified for 10−11 cycles. The amplified product was visualized on an Agilent DNA1000 chip or Caliper HiSens Bioanalyzer assay. Libraries were pooled at equimolar concentrations based on these results and size selected using a Sage PippinPrep 2% cassette. The final library pool had an average insert size of 170 bp with ~25−30 bp partial overlap between pairs of reads. It was quantified using a Kapa Biosystems qPCR library quantification kit, then sequenced on the Illumina HiSeq1000 in a 2 × 101 paired-end sequencing run using dedicated read indexing. The samples were demultiplexed with CASAVA 1.8.2. Details on the library output are given in Table S4. Further information about sequencing datasets and contextual data are available at PANGAEA [61, 82].

### Ribosomal and metabolic gene reconstruction from metagenomic data

16S rRNA and metabolic gene abundances as well as gene reconstructions were generated using a novel, modified version of the phyloFlash pipeline (https://github.com/HRGV/phyloFlash) called funcFlash for metabolic genes. In brief, the generated reads were mapped with BBMap at minimal global nucleotide identity of 70% against curated nucleotide databases: The SILVA SSURef v119 database [75], a published *dsrAB* gene database (http://www.microbial-ecology.net/db_download/dsr_v3.zip, [83]) and two newly generated *pmoA* and *mcrA* gene databases that are publicly available at PANGAEA; see ref. [61]. The mapped read pairs were counted when at least one read had a positive mapping. Full-length (>70% of the target length) genes were assembled with SPAdes [84] or reconstructed with EMIRGE [85]. The mapping of reconstructed metabolic genes to curated databases using funcFlash can be used to distinguish, whether a gene of interest is affiliated with organisms performing the reductive or oxidative pathway. In our case this new analysis allowed us to distinguish between *dsrAB* genes from sulfate reducers and from sulfur oxidizers, as well as between *mcrA* genes from methanogens and from methanotrophs.

### Multivariate analyses of metabolic gene families from metagenomic data

We used metagenomic data from seven sites to investigate richness, abundance, and turnover of gene families across the different zones of HMMV. Each metagenome was filtered to remove low-quality and/or short reads. Raw reads were merged into paired reads with BBMerge [86]. To enable the comparison of gene diversity across sites, each metagenome was subsampled to 10^6^ paired reads using the BBMap “reformat” tool. Subsampled reads were analyzed with humann2 [87]. Here, the reads were subjected to a translated nucleotide search against species-level clusters of nonredundant gene families of the UniRef50 database [88] using DIAMOND [89]. Gene abundances were normalized according to gene length, and then conjoined to obtain a *gene* *×* *metagenome* table, analogous to an *OTU ×* *sample* table, with gene families as rows and metagenomes as columns. This matrix allowed us to investigate diversity using metabolic gene families (functions) rather than the commonly used ribosomal genes (taxonomy). The *gene* *×* *metagenome* table was subjected to multivariate analyses (data reduction, hypothesis testing, visualization) based on the R packages *vegan*, *labdsv*, *UpSetR* [90] and customized R scripts. Prior to the analyses we removed gene families with less than ten read hits cumulated across all metagenomes, to focus on abundant gene families and to minimize the influence of spurious hits. NMDS ordinations, calculated percentages of shared gene families and the UpsetR diagram are based on a presence/absence matrix. Diversity indices and rarefaction curves were calculated with a subsampling-based iterative approach using abundance information. Refer to SI for details.

### Nucleotide sequence accession and contextual data availability

16S rRNA amplicon and shotgun metagenomic data are publicly available under SRA Bioproject PRJNA248084 (https://www.ncbi.nlm.nih.gov/bioproject/PRJNA248084/). Gene sequences were archived under accession numbers KX581156-KX581194 (16S rRNA), KX581122-KX581155 (mcrA) and KX581195-KX581216 (pmoA). Comprehensive contextual data [61] are publicly available from the publisher for Earth and environmental data PANGAEA under (https://doi.pangaea.de/10.1594/PANGAEA.861266).

### Cell counts and catalyzed reporter deposition fluorescence in situ hybridization (CARD-FISH)

Total numbers of single cells were determined using acridine orange direct counts according to the protocol published elsewhere [91]. CARD-FISH was performed as previously described [54] with the following modifications. 4−6 µl of 25-fold diluted sediment were used for filtration. Archaeal cell walls were permeabilized with 0.1 M HCl for 2 min to detect ANME-3 cells, or Proteinase K solution (15 µg ml^−1^ (Merck, Darmstadt, Germany) in 0.05 M EDTA (pH 8), 0.1 M Tris-HCl (pH 8), 0.5 M NaCl) for 2−4 min at room temperature for all other archaea. Bacterial cell walls were permeabilized with lysozyme solution (1000 kU/ml) for 60 min at 37 °C. Cells were stained with DAPI (1 µg/ml), embedded in mounting medium and counted in 40−60 independent microscopic fields using an Axiophot II epifluorescence microscope (Carl Zeiss, Jena, Germany). Cell numbers of dense aggregates were estimated semi-quantitatively as previously described [47]. A complete list of oligonucleotide probes, helpers, and competitors used in this study is provided (Table S3). A summary of the results from all 260 CARD-FISH experiments is publicly available at PANGAEA; see ref. [61].

## Electronic supplementary material

Supplemental Material

## References

[1] Reeburgh W (2007). Oceanic methane biogeochemistry. Chem Rev.

[2] Ruff SE, Biddle JF, Teske AP, Knittel K, Boetius A, Ramette A (2015). Global dispersion and local diversification of the methane seep microbiome. Proc Natl Acad Sci USA.

[3] Tavormina PL, Ussler W, Orphan VJ (2008). Planktonic and sediment-associated aerobic methanotrophs in two seep systems along the North American margin. Appl Environ Microbiol.

[4] Bessette S, Moalic Y, Gautey S, Lesongeur F, Godfroy A, Toffin L (2017). Relative abundance and diversity of bacterial methanotrophs at the oxic–anoxic interface of the Congo deep-sea fan. Front Microbiol.

[5] Paul BG, Ding H, Bagby SC, Kellermann MY, Redmond MC, Andersen GL (2017). Methane-oxidizing bacteria shunt carbon to microbial mats at a marine hydrocarbon seep. Front Microbiol.

[6] Knittel K, Boetius A (2009). Anaerobic oxidation of methane: progress with an unknown process. Annu Rev Microbiol.

[7] Orphan VJ, House CH, Hinrichs KU, McKeegan KD, DeLong EF (2002). Multiple archaeal groups mediate methane oxidation in anoxic cold seep sediments. Proc Natl Acad Sci USA.

[8] Bhattarai S, Cassarini C, Gonzalez-Gil G, Egger M, Slomp CP, Zhang Y (2017). Anaerobic methane-oxidizing microbial community in a coastal marine sediment: anaerobic methanotrophy dominated by ANME-3. Microb Ecol.

[9] Winkel M, Mitzscherling J, Overduin PP, Horn F, Winterfeld M, Rijkers R (2018). Anaerobic methanotrophic communities thrive in deep submarine permafrost. Sci Rep.

[10] Ruff SE, Kuhfuss H, Wegener G, Lott C, Ramette A, Wiedling J (2016). Methane seep in shallow-water permeable sediment harbors high diversity of anaerobic methanotrophic communities, Elba, Italy. Front Microbiol.

[11] Wasmund K, Kurtböke DI, Burns KA, Bourne DG (2009). Microbial diversity in sediments associated with a shallow methane seep in the tropical Timor Sea of Australia reveals a novel aerobic methanotroph diversity. FEMS Microbiol Ecol.

[12] Boetius A, Wenzhöfer F (2013). Seafloor oxygen consumption fuelled by methane from cold seeps. Nat Geosci.

[13] Joye SB, Bowles MW, Samarkin VA, Hunter KS, Niemann H (2010). Biogeochemical signatures and microbial activity of different cold-seep habitats along the Gulf of Mexico deep slope. Deep Res Part II Top Stud Oceanogr.

[14] Niemann H, Lösekann T, de Beer D, Elvert M, Nadalig T, Knittel K (2006). Novel microbial communities of the Haakon Mosby mud volcano and their role as a methane sink. Nature.

[15] Felden J, Wenzhöfer F, Feseker T, Boetius A (2010). Transport and consumption of oxygen and methane in different habitats of the Håkon Mosby Mud Volcano (HMMV). Limnol Oceanogr.

[16] Andreassen K, Hubbard A, Winsborrow M, Patton H, Vadakkepuliyambatta S, Plaza-Faverola A (2017). Massive blow-out craters formed by hydrate-controlled methane expulsion from the Arctic seafloor. Science (80-).

[17] Dickens GR (2003). Rethinking the global carbon cycle with a large, dynamic and microbially mediated gas hydrate capacitor. Earth Planet Sci Lett.

[18] Kessler JD, Valentine DL, Redmond MC, Du M, Chan EW, Mendes SD (2011). A persistent oxygen anomaly reveals the fate of spilled methane in the Deep Gulf of Mexico. Science (80-).

[19] Crespo-Medina M, Meile CD, Hunter KS, Diercks AR, Asper VL, Orphan VJ (2014). The rise and fall of methanotrophy following a deepwater oil-well blowout. Nat Geosci.

[20] Joye SB, Teske AP, Kostka JE (2014). Microbial dynamics following the Macondo Oil Well blowout across Gulf of Mexico environments. Bioscience.

[21] Kopf AJ (2002). Significance of mud volcanism. Rev Geophys.

[22] Milkov AV, Sassen R, Apanasovich TV, Dadashev FG (2003). Global gas flux from mud volcanoes: a significant source of fossil methane in the atmosphere and the ocean. Geophys Res Lett.

[23] De Beer D, Sauter E, Niemann H, Kaul N, Foucher JP, Witte U (2006). In situ fluxes and zonation of microbial activity in surface sediments of the Håkon Mosby mud volcano. Limnol Oceanogr.

[24] Felden J, Lichtschlag A, Wenzhöfer F, de Beer D, Feseker T, Pop Ristova P (2013). Limitations of microbial hydrocarbon degradation at the Amon mud volcano (Nile deep-sea fan). Biogeosciences.

[25] Feseker T, Boetius A, Wenzhöfer F, Blandin J, Olu K, Yoerger DR (2014). Eruption of a deep-sea mud volcano triggers rapid sediment movement. Nat Commun.

[26] Pickett Steward T. A. (1989). Space-for-Time Substitution as an Alternative to Long-Term Studies. Long-Term Studies in Ecology.

[27] Marcon Y. Georeferenced photomosaic of the Håkon Mosby mud volcano during Maria S. Merian cruise MSM16/2 (LOOME), link to GeoTIFF archive (32 GB). 2016. 10.1594/PANGAEA.864702

[28] Milkov AV, Vogt PR, Crane K, Lein AY, Sassen R, Cherkashev GA (2004). Geological, geochemical, and microbial processes at the hydrate-bearing Haakon Mosby mud volcano: a review. Chem Geol.

[29] Vogt RP, Gardner J, Crane K (1999). The Norwegian–Barents–Svalbard (NBS) continental margin: introducing a natural laboratory of mass wasting, hydrates, and ascent of sediment, pore water, and methane. Geo-Mar Lett.

[30] Foucher JP, Dupré S, Scalabrin C, Feseker T, Harmegnies F, Nouzé H (2010). Changes in seabed morphology, mud temperature and free gas venting at the Håkon Mosby mud volcano, offshore northern Norway, over the time period 2003-6. Geo-Mar Lett.

[31] Perez-Garcia C, Feseker T, Mienert J, Berndt C (2009). The Håkon Mosby mud volcano: 330 000 years of focused fluid flow activity at the SW Barents Sea slope. Mar Geol.

[32] Lein A, Vogt P, Crane K, Egorov A, Ivanov M (1999). Chemical and isotopic evidence for the nature of the fluid in CH4-containing sediments of the Håkon Mosby Mud Volcano. Geo-Mar Lett.

[33] Luff R, Wallmann K (2003). Fluid flow, methane fluxes, carbonate precipitation and biogeochemical turnover in gas hydrate-bearing sediments at Hydrate Ridge, Cascadia Margin: numerical modeling and mass balances. Geochim Cosmochim Acta.

[34] Joye SB, Boetius A, Orcutt BN, Montoya JP, Schulz HN, Erickson MJ (2004). The anaerobic oxidation of methane and sulfate reduction in sediments from Gulf of Mexico cold seeps. Chem Geol.

[35] Bowles M. W., Mogollon J. M., Kasten S., Zabel M., Hinrichs K.-U. (2014). Global rates of marine sulfate reduction and implications for sub-sea-floor metabolic activities. Science.

[36] Feseker T, Foucher JP, Harmegnies F (2008). Fluid flow or mud eruptions? Sediment temperature distributions on Håkon Mosby mud volcano, SW Barents Sea slope. Mar Geol.

[37] Parkes RJ, Cragg B, Roussel E, Webster G, Weightman A, Sass H (2014). A review of prokaryotic populations and processes in sub-seafloor sediments, including biosphere:geosphere interactions. Mar Geol.

[38] Blazejak A, Schippers A (2010). High abundance of JS-1- and Chloroflexi-related Bacteria in deeply buried marine sediments revealed by quantitative, real-time PCR. FEMS Microbiol Ecol.

[39] Nobu MK, Narihiro T, Rinke C, Kamagata Y, Tringe SG, Woyke T (2015). Microbial dark matter ecogenomics reveals complex synergistic networks in a methanogenic bioreactor. ISME J.

[40] Lloyd KG, Schreiber L, Petersen DG, Kjeldsen KU, Lever MA, Steen AD (2013). Predominant archaea in marine sediments degrade detrital proteins. Nature.

[41] Hoshino T, Toki T, Ijiri A, Morono Y, Machiyama H, Ashi J (2017). Atribacteria from the subseafloor sedimentary biosphere disperse to the hydrosphere through submarine mud volcanoes. Front Microbiol.

[42] Durbin AM, Teske A (2011). Microbial diversity and stratification of South Pacific abyssal marine sediments. Environ Microbiol.

[43] Braun S, Mhatre SS, Jaussi M, Røy H, Kjeldsen KU, Pearce C (2017). Microbial turnover times in the deep seabed studied by amino acid racemization modelling. Sci Rep.

[44] Starnawski P, Bataillon T, Ettema TJG, Jochum LM, Schreiber L, Chen X (2017). Microbial community assembly and evolution in subseafloor sediment. Proc Natl Acad Sci USA.

[45] Trembath-Reichert E, Morono Y, Ijiri A, Hoshino T, Dawson KS, Inagaki F (2017). Methyl-compound use and slow growth characterize microbial life in 2-km-deep subseafloor coal and shale beds. Proc Natl Acad Sci USA.

[46] Lichtschlag A, Felden J, Brüchert V, Boetius A, de Beer D (2010). Geochemical processes and chemosynthetic primary production in different thiotrophic mats of the Haakon Mosby Mud Volcano (Barents Sea). Limnol Oceanogr.

[47] Lösekann T, Knittel K, Nadalig T, Fuchs B, Niemann H, Boetius A (2007). Diversity and abundance of aerobic and anaerobic methane oxidizers at the Haakon Mosby Mud Volcano, Barents Sea. Appl Environ Microbiol.

[48] Nauhaus K, Albrecht M, Elvert M, Boetius A, Widdel F (2007). In vitro cell growth of marine archaeal−bacterial consortia during anaerobic oxidation of methane with sulfate. Environ Microbiol.

[49] Hatzenpichler R, Connon SA, Goudeau D, Malmstrom RR, Woyke T, Orphan VJ (2016). Visualizing in situ translational activity for identifying and sorting slow-growing archaeal−bacterial consortia. Proc Natl Acad Sci USA.

[50] Girguis PR, Cozen AE, DeLong EF (2005). Growth and population dynamics of anaerobic methane-oxidizing archaea and sulfate-reducing bacteria in a continuous-flow bioreactor. Appl Environ Microbiol.

[51] Marlow J, Skennerton CT, Li Z, Chourey K, Hettich R, Pan C (2016). Proteomic stable isotope probing reveals biosynthesis dynamics of slow growing methane based microbial communities. Front Microbiol.

[52] Tavormina PL, Ussler W, Joye SB, Harrison BK, Orphan VJ (2010). Distributions of putative aerobic methanotrophs in diverse pelagic marine environments. ISME J.

[53] Dedysh SN, Panikov NS, Liesack W, Grosskopf R, Zhou J, Tiedje JM (1998). Isolation of acidophilic methane-oxidizing bacteria from northern peat wetlands. Science (80-).

[54] Ruff SE, Arnds J, Knittel K, Amann R, Wegener G, Ramette A (2013). Microbial communities of deep-sea methane seeps at Hikurangi Continental Margin (New Zealand). PLoS ONE.

[55] Sommer S, Linke P, Pfannkuche O, Niemann H, Treude T (2010). Benthic respiration in a seep habitat dominated by dense beds of ampharetid polychaetes at the Hikurangi Margin (New Zealand). Mar Geol.

[56] Galand PE, Casamayor EO, Kirchman DL, Lovejoy C (2009). Ecology of the rare microbial biosphere of the Arctic Ocean. Proc Natl Acad Sci USA.

[57] Jousset A, Bienhold C, Chatzinotas A, Gallien L, Gobet A, Kurm V (2017). Where less may be more: how the rare biosphere pulls ecosystems strings. ISME J.

[58] Goffredi SK, Wilpiszeski R, Lee R, Orphan VJ (2008). Temporal evolution of methane cycling and phylogenetic diversity of archaea in sediments from a deep-sea whale-fall in Monterey Canyon, California. ISME J.

[59] Bienhold C, Pop Ristova P, Wenzhöfer F, Dittmar T, Boetius A (2013). How deep-sea Wood Falls sustain chemosynthetic life. PLoS ONE.

[60] Pop Ristova P, Bienhold C, Wenzhöfer F, Rossel PE, Boetius A (2017). Temporal and spatial variations of bacterial and faunal communities associated with deep-sea Wood Falls. PLoS ONE.

[61] Ruff SE, Felden J, Marcon Y, Ramette A, Boetius A. Development of bacterial and archaeal communities in erupted subsurface muds at the Håkon Mosby mud volcano. 2016. https://doi.pangaea.de/10.1594/PANGAEA.861266

[62] Felden J, Ruff SE, Ertefai T, Inagaki F, Hinrichs KU, Wenzhöfer F (2014). Anaerobic methanotrophic community of a 5346-m-deep vesicomyid clam colony in the Japan Trench. Geobiology.

[63] Cline JD (1969). Spectrophotometric determination of hydrogen sulfide in natural waters. Limnol Oceanogr.

[64] Hall POJ, Aller RC (1992). Rapid, small-volume, flow injection analysis for ΣCO2 and NH4+in marine and freshwaters. Limnol Oceanogr.

[65] Grasshoff K, Kremling K, Ehrhardt M (1999). Methods of seawater analysis.

[66] Jørgensen BB (1978). A comparison of methods for the quantification of bacterial sulfate reduction in coastal marine sediments. Geomicrobiol J.

[67] Treude T, Boetius A, Knittel K, Wallmann K, Jørgensen BB (2003). Anaerobic oxidation of methane above gas hydrates at Hydrate Ridge, NE Pacific Ocean. Mar Ecol Prog Ser.

[68] Kallmeyer J, Ferdelman TG, Weber A, Fossing H, Jørgensen BB (2004). A cold chromium distillation procedure for radiolabeled sulfide applied to sulfate reduction measurements. Limnol Oceanogr Methods.

[69] Schloss PD, Westcott SL, Ryabin T, Hall JR, Hartmann M, Hollister EB (2009). Introducing mothur: open-source, platform-independent, community-supported software for describing and comparing microbial communities. Appl Environ Microbiol.

[70] Schloss PD, Gevers D, Westcott SL (2011). Reducing the effects of PCR amplification and sequencing artifacts on 16S rRNA-based studies. PLoS ONE.

[71] Quince C, Lanzen A, Curtis TP, Davenport RJ, Hall N, Head IM (2009). Accurate determination of microbial diversity from 454 pyrosequencing data. Nat Methods.

[72] Huse SM, Welch DM, Morrison HG, Sogin ML (2010). Ironing out the wrinkles in the rare biosphere through improved OTU clustering. Environ Microbiol.

[73] Edgar RC, Haas BJ, Clemente JC, Quince C, Knight R (2011). UCHIME improves sensitivity and speed of chimera detection. Bioinformatics.

[74] Yarza P, Yilmaz P, Pruesse E, Glöckner FO, Ludwig W, Schleifer KH (2014). Uniting the classification of cultured and uncultured bacteria and archaea using 16S rRNA gene sequences. Nat Rev Microbiol.

[75] Quast C, Pruesse E, Yilmaz P, Gerken J, Schweer T, Yarza P (2013). The SILVA ribosomal RNA gene database project: improved data processing and web-based tools. Nucleic Acids Res.

[76] Chao A (1984). Nonparametric estimation of the number of classes in a population. Scand J Stat.

[77] Bray JR, Curtis JT (1957). An ordination of the Upland forest communities of Southern Wisconsin. Ecol Monogr.

[78] Kruskal JB (1964). Nonmetric multidimensional scaling: a numerical method. Psychometrika.

[79] Oksanen J, Blanchet FG, Kindt R, Legendre P, Minchin PR, O´Hara RB, et al. vegan: Community Ecology Package. 2012. https://CRAN.R-project.org/package=vegan

[80] Roberts DW labdsv: Ordination and multivariate analysis for ecology. 2012. https://CRAN.R-project.org/package=labdsv

[81] Zhou J, Bruns MA, Tiedje JM (1996). DNA recovery from soils of diverse composition. Appl Environ Microbiol.

[82] Ruff SE, Ramette A, Boetius A. Metadata und statistical analysis of archaeal and bacterial sequences originating from sediments of the Håkon Mosby mud volcano. 2016. https://doi.pangaea.de/10.1594/PANGAEA.861873

[83] Müller AL, Kjeldsen KU, Rattei T, Pester M, Loy A (2015). Phylogenetic and environmental diversity of DsrAB-type dissimilatory (bi)sulfite reductases. ISME J.

[84] Bankevich A, Nurk S, Antipov D, Gurevich AA, Dvorkin M, Kulikov AS (2012). SPAdes: a new genome assembly algorithm and its applications to single-cell sequencing. J Comput Biol.

[85] Miller CS, Baker BJ, Thomas BC, Singer SW, Banfield JF (2011). EMIRGE: reconstruction of full-length ribosomal genes from microbial community short read sequencing data. Genome Biol.

[86] Bushnell B, Rood J, Singer E (2017). BBMerge—accurate paired shotgun read merging via overlap. PLoS ONE.

[87] Abubucker S, Segata N, Goll J, Schubert AM, Izard J, Cantarel BL (2012). Metabolic reconstruction for metagenomic data and its application to the human microbiome. PLoS Comput Biol.

[88] Suzek BE, Wang Y, Huang H, McGarvey PB, Wu CH, Consortium the U. (2015). UniRef clusters: a comprehensive and scalable alternative for improving sequence similarity searches. Bioinformatics.

[89] Buchfink B, Xie C, Huson DH (2014). Fast and sensitive protein alignment using DIAMOND. Nat Methods.

[90] Conway JR, Lex A, Gehlenborg N (2017). UpSetR: an R package for the visualization of intersecting sets and their properties. bioRxiv.

[91] Meyer-Reil LA (1983). Benthic response to sedimentation events during autumn to spring at a shallow water station in the Western Kiel Bight. Mar Biol.

