# Supplementary material for: In situ development of a methanotrophic microbiome in deep-sea sediments
Source: ISME J. 2018 Aug 28;13(1):197–213. doi: 10.1038/s41396-018-0263-1 (PMC6298960; doi:10.1038/s41396-018-0263-1)
Supplement: Supplementary file 1 — Supplemental Material [file 41396_2018_263_MOESM1_ESM.docx]

Supplementing Information for

**In situ development of a methanotrophic microbiome in deep-sea sediments**

S. E. Ruff, J. Felden, H. R. Gruber-Vodicka, Y. Marcon, K. Knittel, A. Ramette, A. Boetius

correspondence to: [emil.ruff@ucalgary.ca](mailto:emil.ruff@ucalgary.ca); antje.boetius@awi.de

**This PDF file includes:**

Figs. S1 to S8

Tables S1 to S6

**Other Supplementing Information for this manuscript includes the following files that are publicly available at PANGAEA** [1]**:**

Ruff_et_al_HMMV_Table_of_Gene_Families.txt (UniRef50-based gene families of all metagenomes)

Ruff_et_al_HMMV_OTU_of_Key_Populations.xlsx (List of operational taxonomic units of key populations)

Ruff_et_al_HMMV_All_OTU_Archaea.txt (List of all archaeal operational taxonomic units)

Ruff_et_al_HMMV_All_OTU_Bacteria.txt (List of all bacterial operational taxonomic units)

Ruff_et_al_ARB_database_mcrA_gene (Curated database of >2k mcrA genes including alignments and phylogenetic trees)

Ruff_et_al_ARB_database_pmoA_gene (Curated database of >2k pmoA genes including alignments and phylogenetic trees)

**Supplementary Materials and Methods**

Habitat mapping by high-resolution photomosaic

A total of 34,118 images were acquired with the AUV Sentry during the M16/2 cruise LOOME (dives 074, 075, 076 and 077). The photos were assembled into a large geo-referenced photomosaic with the LAPM Tool [2]. The absolute geo-referencing of the mosaic was based on the navigation (latitude, longitude, altitude) and attitude (heading, pitch and roll) data of the AUV Sentry. Mats of sulfide-oxidizing bacteria (SOB) could be clearly identified from the images of the mosaic due to the bright whitish color of the mats. Delineation of the SOB patches revealed that their distribution was very variable over the mosaic area. In order to provide quantify the concentration of SOB mats, we calculated their percent coverage for each 1×1 m grid cell of the mosaic (Fig. S1). The full high-resolution photomosaic (32 GB) can be accessed via PANGAEA [3].

Biogeochemistry

The pore water was extracted with Rhizons (Rhizon CSS: length 5 cm, pore diameter 0.15 µm; Rhizosphere Research Products, Wageningen, Netherlands) in 1 cm-resolution and immediately fixed in 5% zinc acetate (ZnAc) solution for sulfate, and sulfide analyses. The samples were diluted, filtered and the concentrations measured with non-suppressed anion exchange chromatography (Waters IC-Pak anion exchange column, waters 430 conductivity detector). The total sulfide concentrations (H_2_S + HS^-^ + S^2-^) were determined using the diamine complexation method [4]. Samples for dissolved inorganic carbon (DIC) and alkalinity measurements were preserved by adding 2 µl saturated mercury chloride (HgCl_2_) solution and stored headspace-free in gas-tight glass vials. DIC and alkalinity were measured using the flow injection method (detector VWR scientific model 1054) [5]. Dissolved sulfide was eliminated prior to the DIC measurement by adding 0.5 M molybdate solution [6]. Nutrient subsamples (10 – 15 ml) were stored at – 20 °C prior to concentration measurements with a Skalar Continuous-Flow Analyzer [7]. Sulfate reduction (SR) and anaerobic oxidation of methane (AOM) were measured *ex situ* by the whole core injection method [8]. We incubated the samples at *in situ* temperature (1.0°C) for 12 hours with either ^14^CH_4_ (dissolved in water, 2.5 kBq) or carrier-free ^35^SO_4_ (dissolved in water, 50 kBq). Sediment was fixed in 25 ml 2.5% sodium hydroxide (NaOH) solution or 20 ml 20% ZnAc solution for AOM or SR, respectively. The ex situ substrate concentrations (methane, sulfate) were measured by gas chromatography (5890A; Hewlett Packard) and anion exchange chromatography (Waters I.C.-PakTM anion column 50 3 4.6 mm; Waters 430 conductivity detector), respectively. Rates were measured as previously described [9, 10]. A summary of all porewater profiles and rate measurements available from the HMMV observatory (2009 and 2010; [1]) and from four additional expeditions (2001, 2003, 2006 and 2007) are presented in Table 2.

Primers for pyrosequencing

We sequenced amplicons spanning multiple variable regions when 454 read lengths increased to 500+. The PCR reactions for V6-V4 employed a pair of degenerate fusion primers (Bacteria: 1064R and 518F; Archaea: 1048R and 517F). The bacterial primer sets cover 96.3 % of the bacterial entries in the SILVA database 132 release [11] as tested with testprime (<https://www.arb-silva.de/search/testprime>), allowing for 1 mismatch. The archaeal primer sets cover >90.7 % of archaeal entries. Further information on sequence datasets, contextual data and sequence analyses are available at PANGAEA [12].

Bacterial v6v4 (454)

- - Forward Primer (518F)  CCAGCAGCYGCGGTAAN
  - Reverse Primer (1064R)  CGACRRCCATGCANCACCT
  - Bioinformatic Trimming anchor site (565F-a)  TGGGCGTAAAG

Archaeal v6v4 (454)

- - Forward Primers (517F) GCCTAAAGCATCCGTAGC,  GCCTAAARCGTYCGTAGC, GTCTAAAGGGTCYGTAGC, GCTTAAAGNGTYCGTAGC, GTCTAAARCGYYCGTAGC
  - Reverse Primer (1048R)   CGRCRGCCATGYACCWC
  - Bioinformatic Trimming anchor site (685F-a)  GWAGRRGTRAAAT

Statistical analyses of amplicons

Stress values of the NMDS ordinations were below 0.2, which indicated that the multidimensional dataset was well represented by the 2D ordination. To test the influence of singletons on the overall community structure we compared NMDS ordinations with and without singletons using the Procrustes correlation analysis *protest* [13]. Since the correlation between both ordinations was highly significant (p=0.001), indicating that singletons did not change the observed differences in community structure between samples, we did not remove the singletons from the dataset. Operational taxonomic units at 98% sequence identity (OTU) that occurred only once in the whole dataset were termed absolute single sequence OTUs (SSO_abs_) [14]. OTU sequences that occurred only once in at least one sample, but may occur more often in other samples were termed relative single sequence OTUs (SSO_rel_) [14]. SSO_rel_ are particularly interesting for community ecology, since they comprise rare organisms that might become abundant when conditions change.

Phylogeny of reconstructed functional genes

Sequences of the metabolic genes *mcrA* and *pmoA* that were reconstructed with funcFlash were translated into amino acids and manually aligned using ARB [15] and custom-made McrA and PmoA databases. Phylogenetic trees were calculated with a 30-100% homology filter using only columns in the amino acid alignment that were complete (McrA: 218 columns, PmoA: 145 columns), a JTT substitution model, the maximum likelihood algorithm phyML and 100 iterations. Short sequences were added using maximum parsimony without changing the overall topology and redundant sequences were removed for clarity.

Multivariate analyses of metabolic gene families from metagenomic data

To integrate metagenomics and community ecology we designed a workflow based on BBTools [16], humann2 [17] and R [18]. Metagenomic reads were merged with BBMerge and filtered based on quality and length. The metagenomes were subsampled to 1 million paired reads using reformat. We then used humann2, a tool for the functional profiling of human microbiomes by the Huttenhower lab (http://huttenhower.sph.harvard.edu/humann2). In a first step, the tool uses bowtie2 [19] to search reads against a custom-built genome database including >4k genomes from NCBI (ChocoPhlAn - provided by the Huttenhower lab). In a second, organism-independent step, the tool uses diamond [20] to search reads against a characterized protein sequence database, in our case Uniref50 clusters of gene families [21]. For each metagenomic sample, humann2 recovers the abundances of individual orthologous gene families by counting its reads’ BLAST hits in a weighted manner, normalized by each gene family’s average sequence length. Finally, the normalized gene abundance tables from each metagenome can be joined to obtain a global table including all genes found in all metagenomes. This *gene × metagenome* table (Ruff_et_al_HMMV_Table_of_Gene_Families.txt) was reformatted to be machine readable and is publicly available at PANGAEA [12]. The table was subjected to an R based community analysis workflow using the packages *vegan* [22], *cluster* [23], *UpSetR* [24], *ggplot2* [25]. Most of the reads of each metagenome – between 57 % (Sample 11) to 72 % of the reads (Sample10) - did not map to any gene family in the UniRef50 database and could therefore not be annotated. These reads may represent hypothetical or unknown genes. The annotated reads still provided ample information and affiliated with over 100k gene families. To not give weight to the unmapped reads and focus on the richness of observed genes we used a presence/absence-based distance matrix for the non-metric multidimensional scaling and the cluster dendrogram. The UpSet diagram – analogous to a Venn diagram – and the percentage of shared genes between metagenomes was also calculated based on the same presence/absence matrix. To calculate diversity indices and rarefaction curves we used the original abundance data, but removed the unmapped reads. We subsampled the *gene × metagenome* table to account for the different proportion of removed reads. Gene richness (number of observed genes), Inverse Simpson evenness, Shannon entropy – also known as Hill numbers - and Chao1 estimated richness were determined as the mean of ten iterated calculations (Table S5).

**Supplementary Results and Discussion**

Previous investigations of the HMMV show a distinct geophysical and geochemical zonation around the active center of the mud volcano, which frequently erupts and transports subsurface muds to the surface of the seabed since decades [26–29]. Visual and acoustic observations, temperature recordings and microbathymetric mapping during expeditions in 2003, 2006, 2009 and 2010 showed that HMMV was highly active in this period, marked by vigorous gas emissions, high fluid flow and heat flux as well as bathymetric and geographical shifts of physical markers and of mapped habitats at the seafloor [30, 31]. The first long-term observation of sediment temperatures from September 2005 to June 2006 yielded evidence of several eruptive events, indicated by abrupt temperature increases of several °C of the seafloor within a few days. High-resolution bathymetric maps and video observations of the seafloor also showed changes in the morphology of HMMV at that time [30]. Generally, across the entire observation period from 2003-2010, the sediment temperature and upward fluid flow decreased from the center of the caldera to its periphery, with 1.3-6 m yr^-1^ in the center (Zone 1, 2) and 0.3-1 m yr^-1^ in Zone 3. The rim around the caldera is stabilized by gas hydrates and populated by dense tubeworm communities (Zone 4; [28, 32]. Seafloor temperatures ranged from 25°C at 50 cm sediment depth in the center to ambient temperature ~0°C in zone 4 [28, 30]. Our previous rate measurements from 2003-2009 showed an unexplained, substantial variation of microbial activity within the HMMV center of around one order of magnitude, which we suggested to originate from fluid flow variations [28]. In 2009 LOOME, an observatory for continuous observation of heat flux, bathymetry and geochemical signatures was deployed [29], which recorded further substantial eruptions in autumn 2009. In 2010 the observations were completed by sampling along a new mud flow that had emerged from the 2009 eruption and had moved southward. Geophysical and geochemical data from 2003 - 2010 [27–30] are available in the environmental data archive PANGAEA [1, 33, 34].

**Supplementary Figures**

**
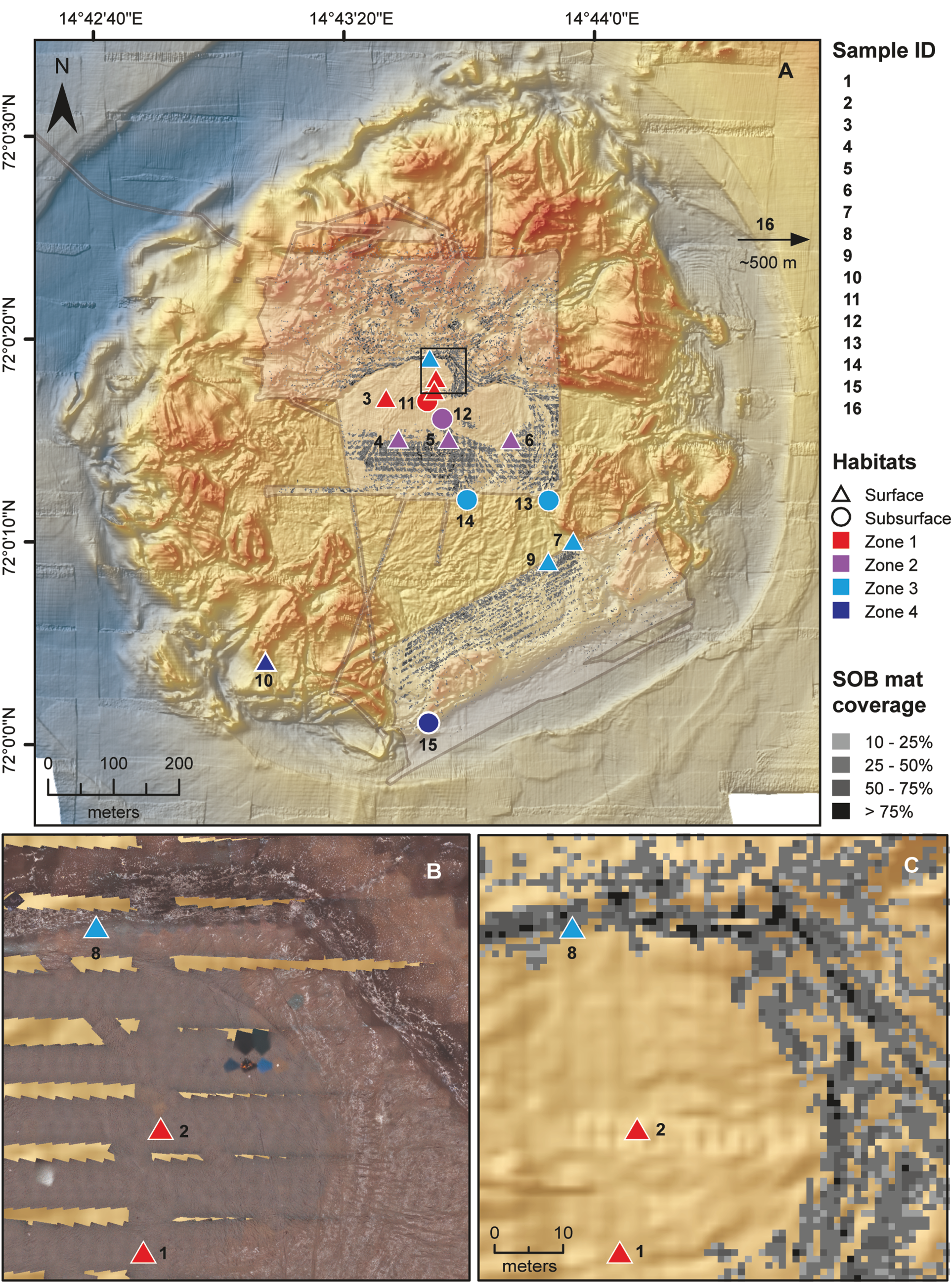
**

Figure_S1_Mosaic-Map_rev2.png

**Fig. S1.** **Map and photomosaic of HMMV** showing the bathymetry, the bacterial mat distribution and the locations of sampling sites (**A**). The transparent, light gray area shows the extent of the photomosaic and the dark gray patches show the areas where the bacterial mat coverage exceeds 10% per 1×1 m grid cell. The black rectangle indicates the area shown in detail (**B**, **C**). The reference site (not shown) is located ~0.5 km outside of the mud volcano structure. **B, C**: Sediment surface at the center of HMMV between the hummocky area and the active area. The photomosaic (**B**) was recorded with AUV Sentry (Woods Hole Oceanographic Institute) and then used to calculate the percentage of sediment that was covered with mats of sulfur-oxidizing bacteria (SOB) (**C**).

**
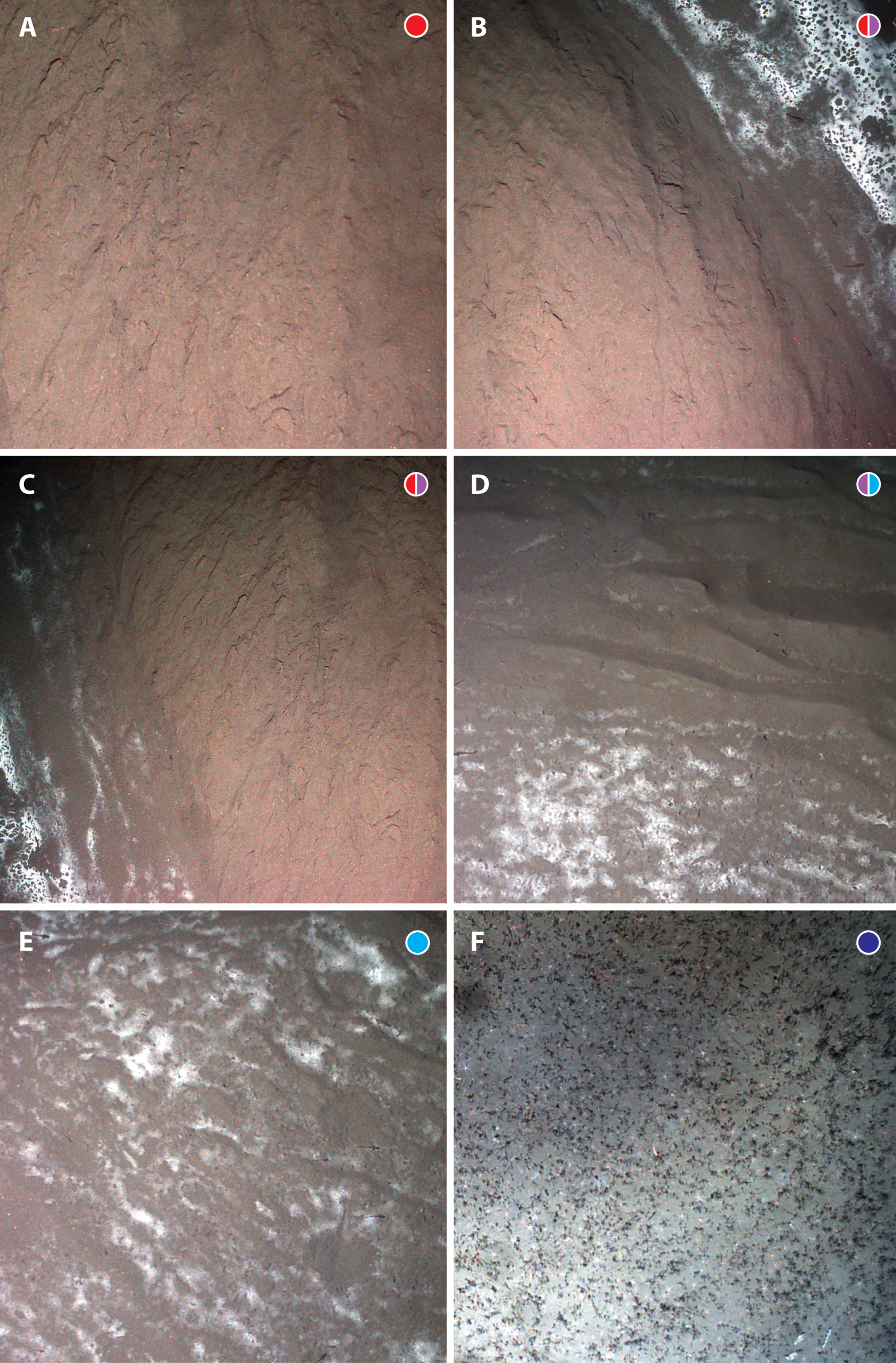
**

**Fig. S2. Seafloor images** showing the surface of sediments from different zones of HMMV. Freshly erupted muds are light brown (**A**) and flow across consolidated sediments that are covered with white mats of sulfur-oxidizing bacteria (**B**, **C** and Fig. 1C (detail of **B**)). Transition between zone 2 and 3 (**D**), consolidated sediments of zone 3 (**E**) and fields of siboglinid tubeworms on the hummocky rim (**F**). Images were taken by AUV Sentry from different locations of the caldera. One image has the size of 4 m by 4 m photographed at 5 m above bottom.

**
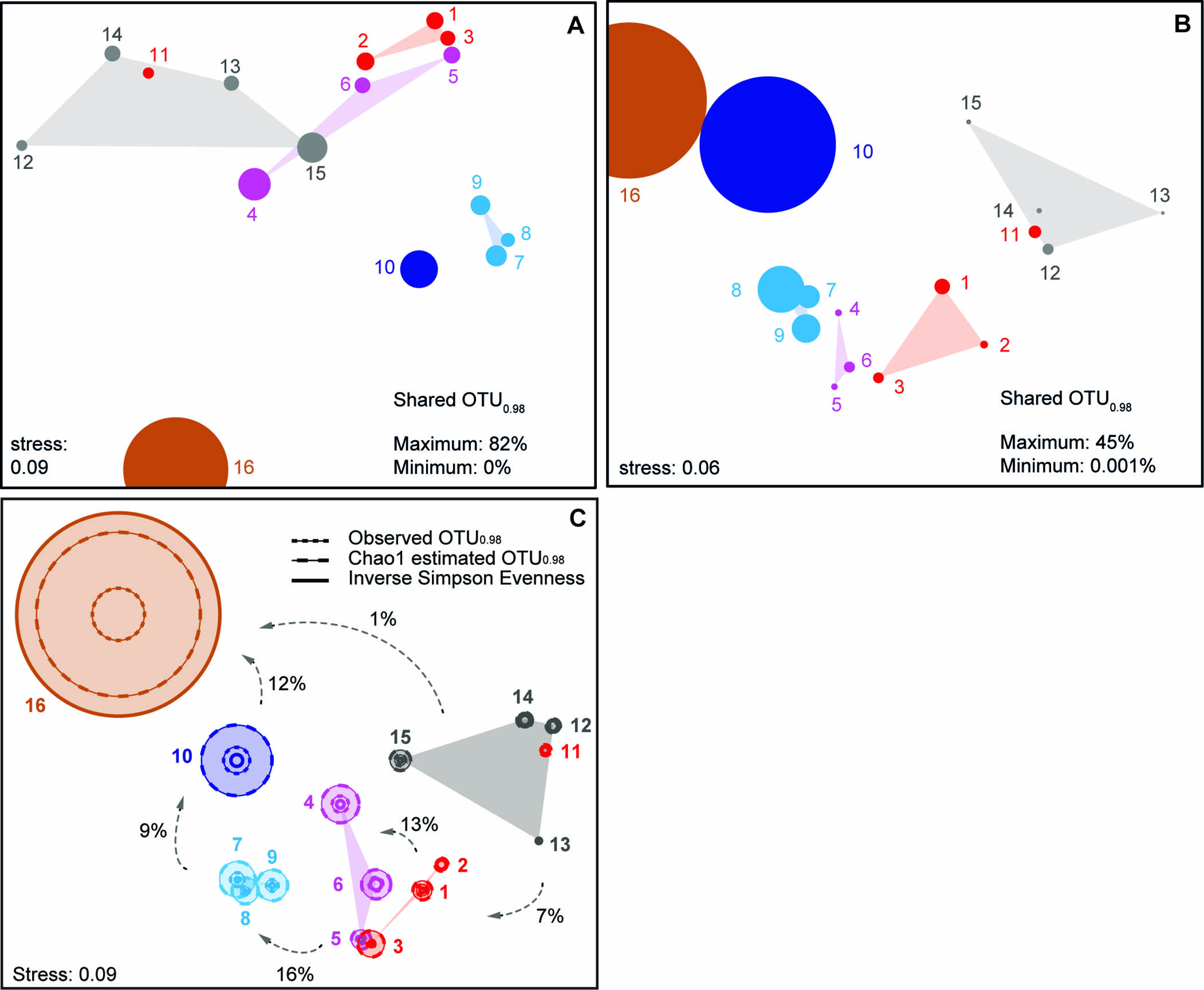
**

**Fig. S3.**

Archaeal (**A**), bacterial (**B**) and total (**C**) community structure of HMMV sediments as visualized by non-metric multi-dimensional scaling (NMDS) of Bray-Curtis dissimilarity matrices based on OTU (operational taxonomic units at 98% 16S rRNA V4-V6 gene sequence identity, corresponding to the recommended taxonomic threshold for microbial species [35]). Color indicates the sample origin (Subsurface = grey; Zone 1 = red; Zone 2 = purple; Zone 3 = light blue; Zone 4 = dark blue; Reference site = brown). Circle area represents the Inverse Simpson diversity index (InvS) of the respective sample (**A**, **B**; Note: Bacterial InvS was divided by 10, in order to visualize both archaeal and bacterial InvS in one figure), or diversity indices as indicated (**C**; values are given in Fig. 2 and Table S1, S2). The archaeal communities of surface muds of zone 1-3 were significantly different from each other (ANOSIM based on presence/absence data: R=0.7, p<0.01). The bacterial community of these zones was different, but overlapping (R=0.5, p<0.01). The total microbial communities of the subsurface and of zone 1-3 were all significantly different from each other (R=0.7, p=0.001). Zone 4 and the reference site could not be included in the ANOSIM as there was only one sample retrieved. The percentages of microbial OTU that are shared between any two sites (**A**, **B**) or between two zones (**C**; numbers next to arrows) are based on presence-absence data, i.e. showing that only 1% of OTU that are present in the subsurface are also found in the seafloor sediment of the reference site (**C**).


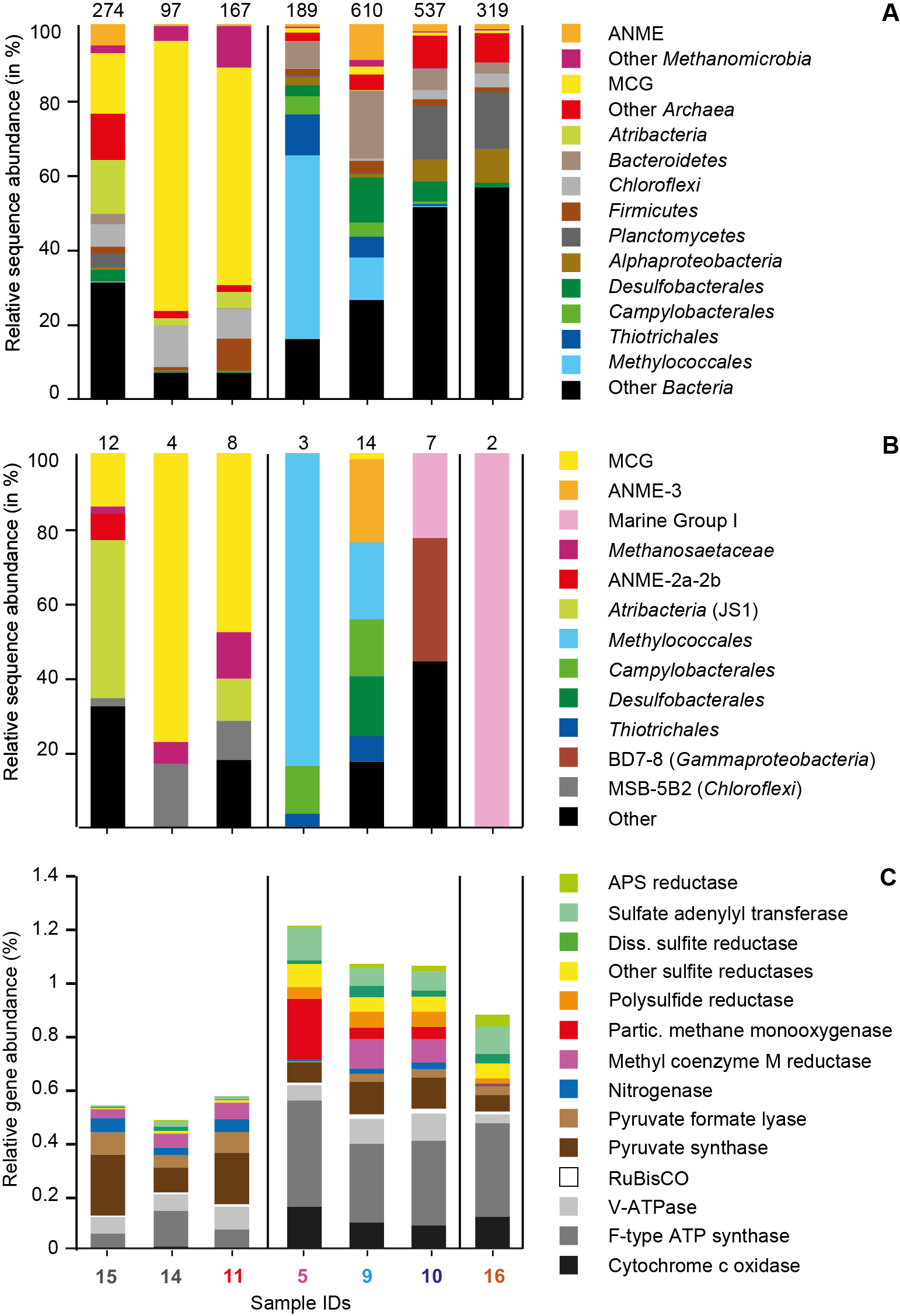


**Fig. S4.**

Microbial community composition and key functions based on metagenomics. **(A)** Archaeal and bacterial relative sequence abundance based on 16S rRNA genes retrieved by mapping of metagenomics reads using the SILVA SSURef database. The number of retrieved operational taxonomic units is given above each column. (**B**) 16S rRNA sequences reconstructed by phyloFlash at very high confidence, the number of sequences per sample is given above the columns. The metagenomics-based microbial diversity corroborates the more detailed diversity patterns that were obtained by pyrosequencing. (**C**) Relative abundances of key metabolic gene families based on the UniRef50 database using humann2 with default settings (a complete list of annotated gene families on species level for all metagenomes is publicly available at PANGAEA, see [12]).


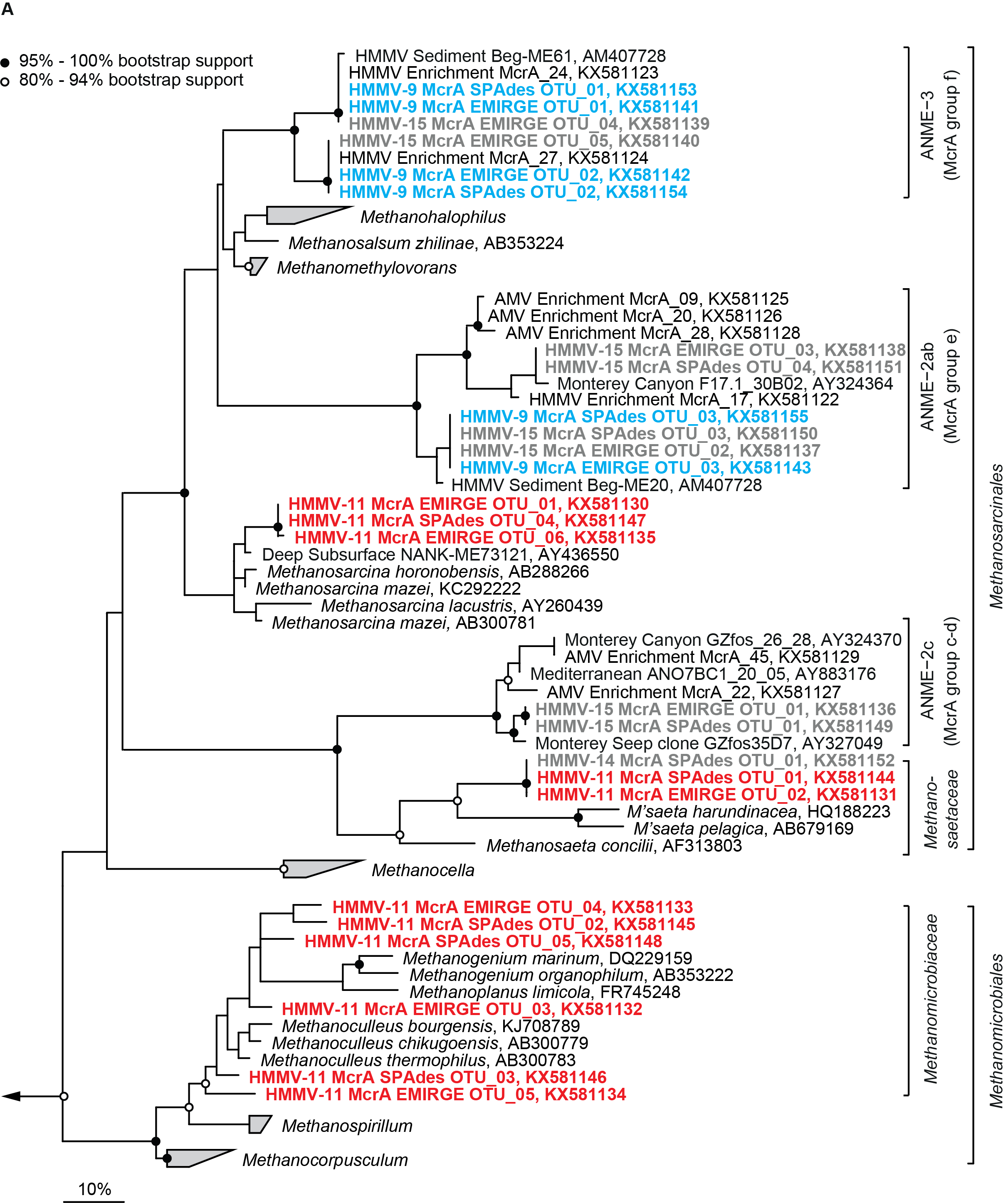


**
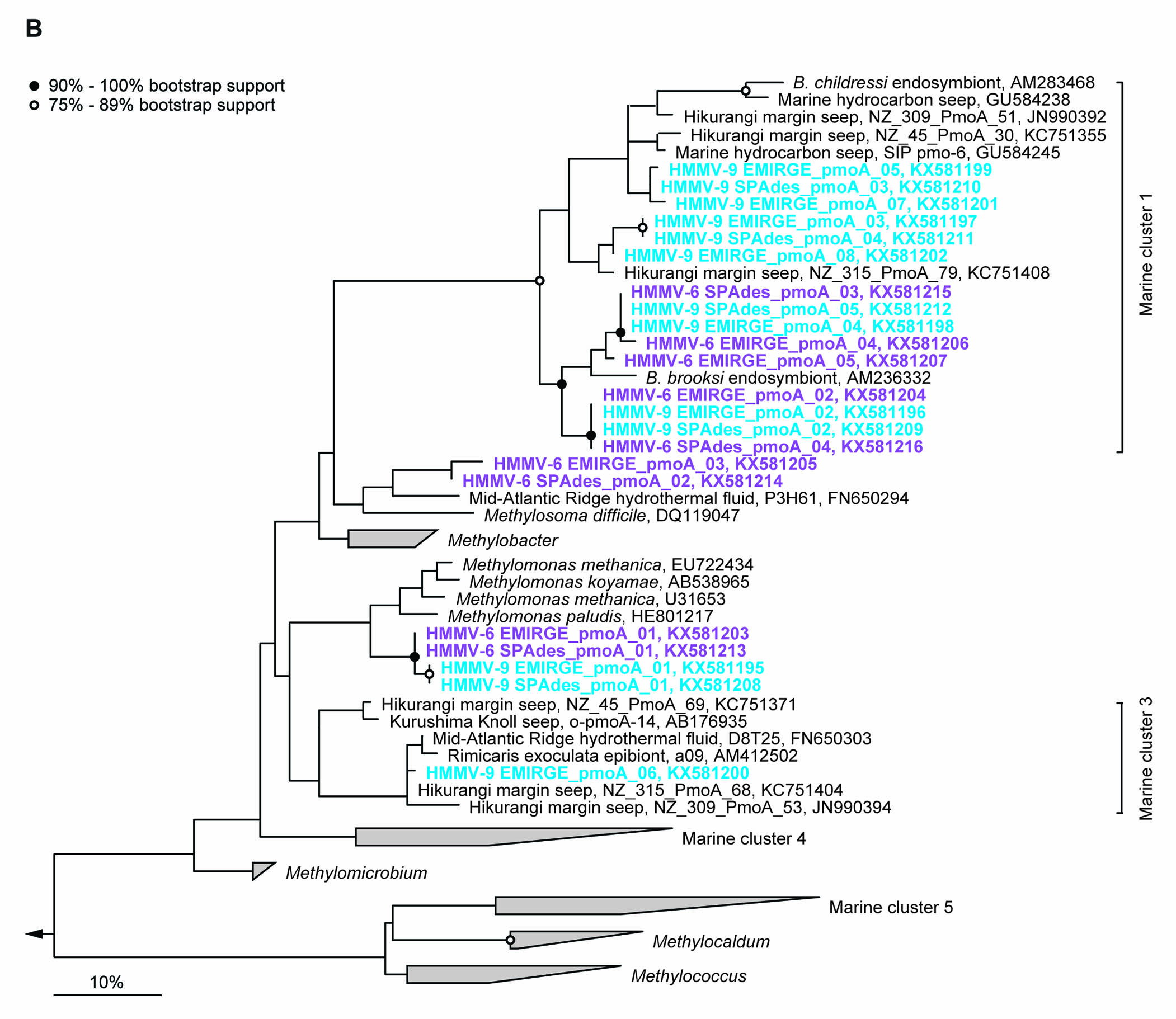
**

**Fig. S5.** **Phylogenetic trees of the methyl coenzyme-M reductase** (McrA) and particulate methane monooxygenase (PmoA) based on aminoacid sequences translated from full length genes that were reconstructed from metagenomes (HMMV-**6**, -**9**, -**11**, -**14**, -**15**). Metabolic genes were reconstructed using the software funcFlash that we specifically developed for this purpose (see Materials & Methods). *Methanomicrobia* (**A**) are archaea and include methanogens and anaerobic methanotrophs, *Methylococcaceae* (**B**) are bacteria and include aerobic methylotrophs. Colors represent the zones of origin (red=subsurface zone 1, purple=surface zone2, light blue=surface zone 3, grey=subsurface zone 2-4). Note: *pmoA* genes were only found in metagenomes of surface sediments. *mcrA* genes affiliating with methanogens exclusively occur in subsurface sediments of the caldera, while *mcrA* genes affiliating with methanotrophs exclusively occur in surface sediments of zone 3 and in the subsurface of the hummocky rim (zone 4), which both are hotspots of AOM. The trees were calculated using phyML as implemented in the software ARB, after manual curation and alignment of gene sequences. Bar depicts estimated sequence divergence.


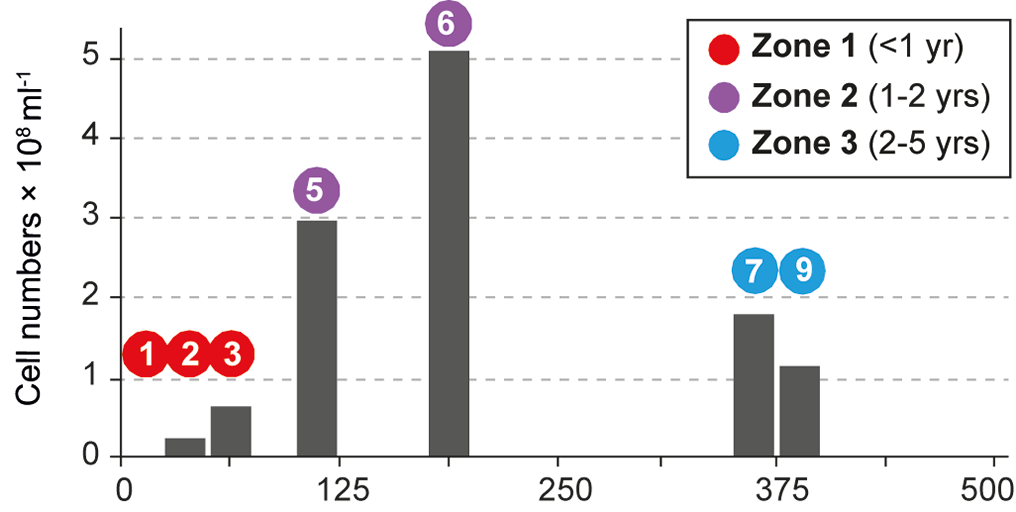


**Fig. S6.** Total cell numbers of *Methylococcales* (probe: MTMC-701) per ml sediment averaged over the top 10 cm sediment depth, showing a tenfold increase in cell numbers during approximately 6 months, which corresponds to a doubling time of 60 days (growth rate: 0.01 day^-1^).

**
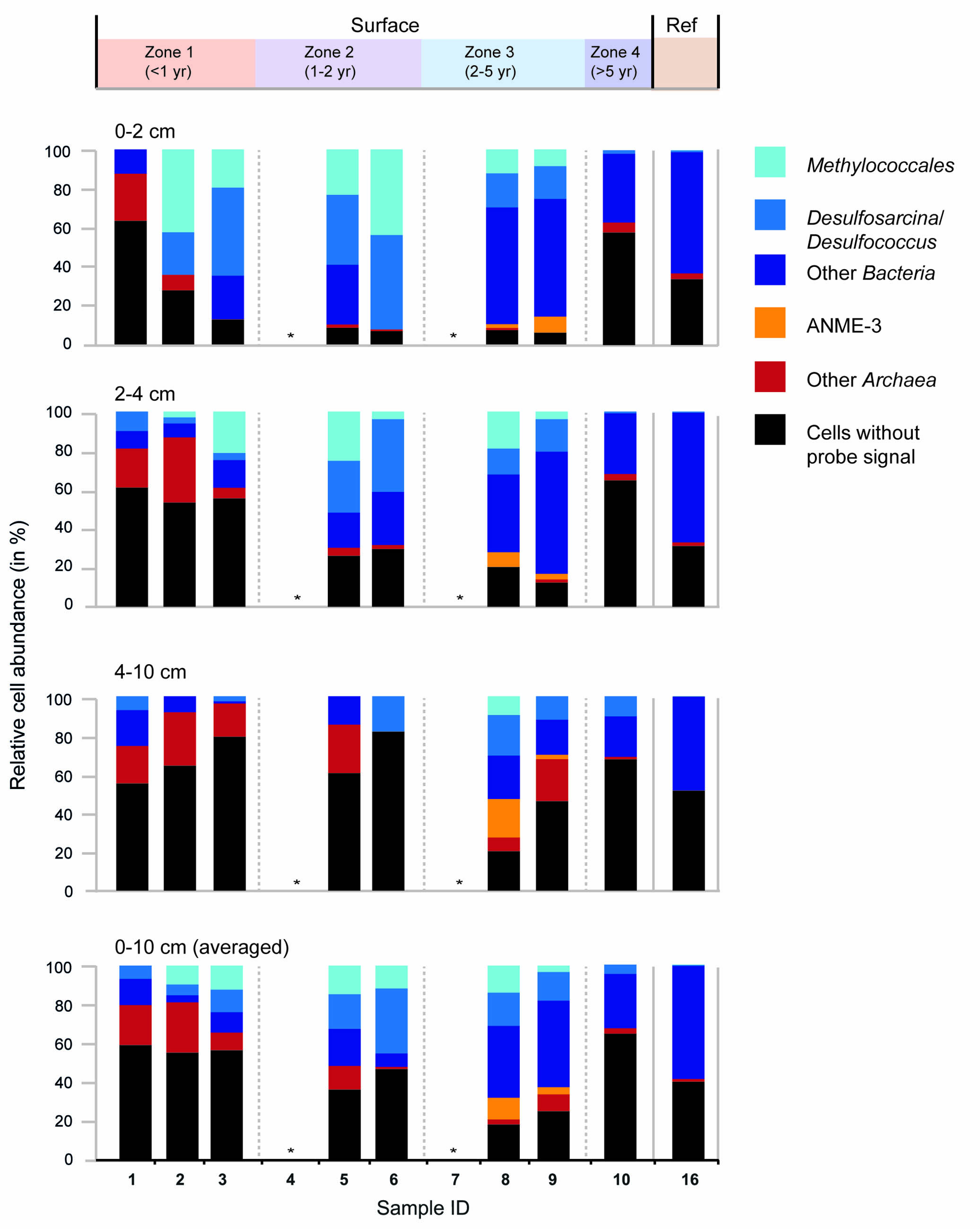
**

**Fig. S7: Relative cell abundances** in surface layers of HMMV based on single cell counts using CARD-FISH with specific probes for *Bacteria* (probe EUB338 I-III), *Methylococcales* (probe MTMC-701 and competitor probes), *Desulfosarcina/Desulfococcus* (probe DSS658), *Archaea* (probe Arch915) and ANME-3 (probe ANME3-1249 and helper probes). *Bacteria* that did not overlap with *Methylococcales* or DSS are denoted “other *Bacteria*”*.* *Archaea* that did not overlap with ANME-3 are referred to as “other *Archaea*”. “Cells without probe signal” were only stained by the nucleic acid stain DAPI and not by general archaeal or bacterial probes. Note: The relative cell abundances for ANME-3, *Desulfosarcina/Desulfococcus* and *Methylococcales* are underestimated as these clades formed cell aggregates that were not included in the single cell counts. Probe details are given in Table S3, detailed values and cell counts are available at PANGAEA [36].

**
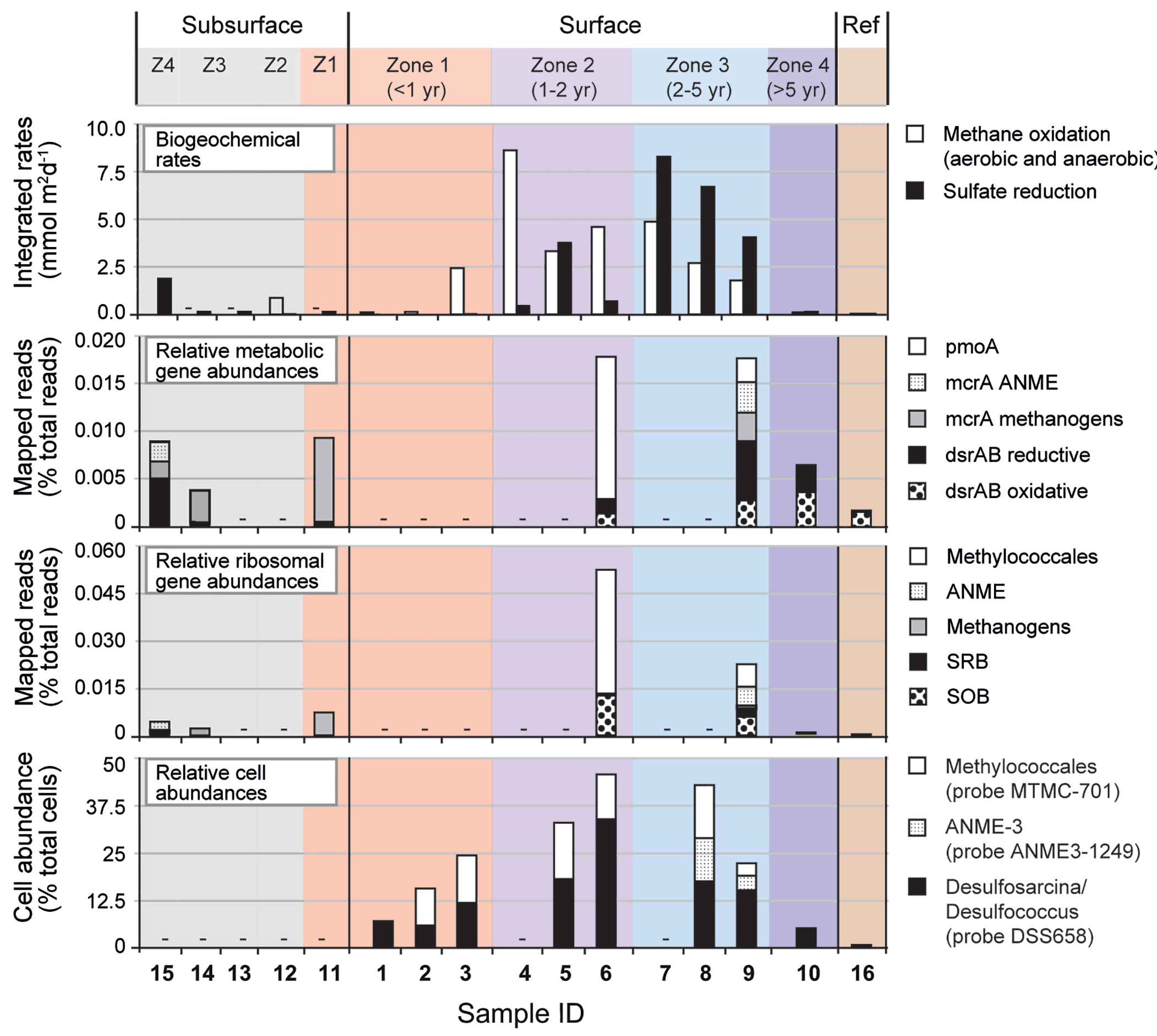
**

**Fig. S8: Methanotrophic rates, relative gene and relative cell abundances.** Rates were measured by tracer incubations and integrated over 0-10 cm sediment depth. Relative gene abundances were obtained by mapping metagenomic reads on a curated database of the respective gene and subsequent normalization. Relative cell abundances were averaged over 0-10 cm sediment depth as determined by CARD-FISH. Dashes denote missing data points.

**Table S1: Diversity measures**

|  | Archaea | | | | |  | Bacteria | | | | |
| --- | --- | --- | --- | --- | --- | --- | --- | --- | --- | --- | --- |
| **Sample**  **ID** | Quality Reads ^#^ | OTU | OTU ^##^ (subsampled) | Chao1 ^###^ Richness | Inverse Simpson Index ^###^ |  | Quality Reads ^#^ | OTU | OTU ^##^ (subsampled) | Chao1 ^###^ Richness | Inverse Simpson Index ^###^ |
| **1** | 15590 | 59 | 10 | 25 | 1.7 |  | 8900 | 404 | 154 | 298 | 13 |
| **2** | 4406 | 22 | 7 | 8 | 1.8 |  | 11522 | 212 | 95 | 156 | 6.6 |
| **3** | 20636 | 70 | 6 | 7 | 1.4 |  | 9732 | 562 | 130 | 288 | 9.6 |
| **4** | 930 | 11 | 6 | 6 | 3.1 |  | 11833 | 569 | 137 | 295 | 5.5 |
| **5** | 23247 | 105 | 12 | 15 | 1.7 |  | 15148 | 589 | 107 | 243 | 6.2 |
| **6** | 4616 | 31 | 11 | 13 | 1.5 |  | 11134 | 475 | 146 | 290 | 9.8 |
| **7** | 37333 | 151 | 8 | 14 | 2.1 |  | 13210 | 1178 | 277 | 707 | 51 |
| **8** | 38457 | 123 | 4 | 4 | 1.2 |  | 10313 | 762 | 191 | 388 | 23 |
| **9** | 30191 | 131 | 11 | 14 | 1.9 |  | 13670 | 900 | 232 | 607 | 27 |
| **10** | 20582 | 153 | 26 | 61 | 4.1 |  | 12637 | 1915 | 433 | 1112 | 116 |
| **11** | 135 | 1 | NA | NA | NA |  | 2718 | 101 | 70 | 154 | 5.7 |
| **12** | 17 | 2 | NA | NA | NA |  | 3138 | 105 | 73 | 115 | 11 |
| **13** | 507 | 10 | 9 | 15 | 1.6 |  | 253 | 22 | NA | NA | NA |
| **14** | 104 | 7 | NA | NA | NA |  | 1542 | 95 | 82 | 107 | 11 |
| **15** | 2446 | 26 | 16 | 18 | 2.9 |  | 7395 | 202 | 101 | 150 | 2.2 |
| **16** | 327 | 50 | 46 | 60 | 11 |  | 14909 | 2975 | 516 | 1755 | 165 |
|  |  |  |  |  |  |  |  |  |  |  |  |
| **^#^** | ^#^ Number of sequences retrieved after processing the data with mothur | | | | | | |  |  |  |  |
| **^##^** | ^##^ Subsampling was performed to account for unequal sampling effort, archaea: 300 sequences, bacteria: 1000 sequences | | | | | | | | | | |
| **^##^** | ^###^ Chao1 Richness and Inverse Simpson Indices were calculated with the full OTU (operational taxonomic units at 98%  16S rRNA V4-V6 gene sequence identity) dataset including singletons | | | | | | | | | |  |

**Table S2: Percentage of shared OTU between HMMV habitats**

**Archaea**

|  | **Subsurface** | **Zone 1** | **Zone 2** | **Zone 3** | **Zone 4** |
| --- | --- | --- | --- | --- | --- |
| **Zone 1** | 16 |  |  |  |  |
| **Zone 2** | 13 | 13 |  |  |  |
| **Zone 3** | 12 | 12 | 11 |  |  |
| **Zone 4** | 10 | 11 | 8 | 11 |  |
| **REF** | 28 | 17 | 14 | 14 | 14 |

**Bacteria**

|  | **Subsurface** | **Zone 1** | **Zone 2** | **Zone 3** | **Zone 4** |
| --- | --- | --- | --- | --- | --- |
| **Zone 1** | 15 |  |  |  |  |
| **Zone 2** | 20 | 16 |  |  |  |
| **Zone 3** | 17 | 15 | 17 |  |  |
| **Zone 4** | 25 | 21 | 23 | 20 |  |
| **REF** | 22 | 20 | 21 | 19 | 23 |

**Archaea and Bacteria**

|  | **Subsurface** | **Zone 1** | **Zone 2** | **Zone 3** | **Zone 4** |
| --- | --- | --- | --- | --- | --- |
| **Zone 1** | 7 |  |  |  |  |
| **Zone 2** | 5 | 13 |  |  |  |
| **Zone 3** | 4 | 8 | 16 |  |  |
| **Zone 4** | 3 | 3 | 6 | 9 |  |
| **REF** | 1 | 2 | 3 | 4 | 12 |

**Table S3: Oligonucleotide probes used in this study**

| **Oligonucleotide** | **Specificity** | **Position on**  **16S rRNA** | **Formamide**  **conc. (% v/v)** | **Nucleotide Sequence 5'-3'** | **Reference** |
| --- | --- | --- | --- | --- | --- |
| ANME-3-1249 | ANME-3 | 1249-1266 | 30 | TCG GAG TAG GGA CCC ATT | [27] |
| ANME-3-1249H3 | Helper | 1249-1266 | 30 | GTC CCA ATC ATT GTA GCC GGC | [37] |
| ANME-3-1249H5 | Helper | 1249-1266 | 30 | TTA TGA GAT TAC CAT CTC CTT | [37] |
| Arch915 | most *Archaea* | 915-934 | 35 | GTG CTC CCC CGC CAA TTC CT | [38] |
| DSS658 | *Desulfosarcina/ Desulfococcus* | 658-675 | 50 | TCC ACT TCC CTC TCC CAT | [39] |
| Eub338-I | most *Bacteria* | 338-355 | 35 | GCT GCC TCC CGT AGG AGT | [40] |
| Eub338-II | Most  *Planctomycetales* | 338-355 | 35 | GCA GCC ACC CGT AGG TGT | [41] |
| Eub338-III | *most Verrucomicrobiales* | 338-355 | 35 | GCT GCC ACC CGT AGG TGT | [41] |
| MTMC701 | Most  *Methylococcales* | 701-718 | 40 | GTG TTC CTT CAG ATC TCT | [42] |
| MTMC701-C1 | Competitor | 701-718 | 40 | GTA TTC CTT CAG ATC TCT | [43] |
| MTMC701-C2 | Competitor | 701-718 | 40 | GTG TTC CTC CAG ATC TCT | [43] |
| NON338 | Nonsense probe | - | 35 | ACT CCT ACG GGA GGC AGC | [44] |

**Table S4: Metagenome specifications**

| Sample  ID | raw PE-reads  (× 10^6^) | insert  size  (bp) | insert size  (sd) | SSU  reads | SSU reads  (%) | dsrAB  reads | dsrAB  reads  (%) | pmoA reads | pmoA  reads  (%) | mcrA reads | mcrA  reads  (%) |
| --- | --- | --- | --- | --- | --- | --- | --- | --- | --- | --- | --- |
| **6** | 8.0 | 175 | 27 | 11702 | 0.15 | 218 | 0.003 | 1190 | 0.0149 | 16 | 0,0002 |
| **9** | 57.3 | 176 | 26 | 64716 | 0.11 | 5153 | 0.009 | 1432 | 0.0025 | 3526 | 0.0062 |
| **10** | 33.3 | 173 | 32 | 17619 | 0.05 | 2106 | 0.006 | 22 | 0.0001 | 29 | 0.0001 |
| **11** | 23.0 | 174 | 26 | 21322 | 0.09 | 124 | 0.001 | 0 | 0 | 2021 | 0.0088 |
| **14** | 10.3 | 174 | 26 | 8596 | 0.08 | 47 | 0.001 | 0 | 0 | 348 | 0.0034 |
| **15** | 20.6 | 171 | 32 | 14348 | 0.07 | 1044 | 0.005 | 19 | 0.0001 | 785 | 0.0038 |
| **16** | 16.1 | 172 | 31 | 7324 | 0.05 | 269 | 0.002 | 10 | 0.0001 | 0 | 0 |

**Table S5: Diversity of gene families based on 1 million subsampled reads**

|  | **Meta-genome ID** | **Number of observed genes*** | **Number of estimated genes (Chao1)** | **Inverse Simpson Index** | **Shannon Entropy** | **Number of SSGabs**** | **Number of SSGrel**** | **SSGabs (%)** | **SSGrel (%)** |
| --- | --- | --- | --- | --- | --- | --- | --- | --- | --- |
| **Including singleton gene families** | **15** | 106384 | 148300 | 42625 | 11.2 | 9110 | 31505 | 7.1 | 24.6 |
|  | **14** | 66408 | 85441 | 13673 | 10.4 | 3017 | 16832 | 3.9 | 22 |
|  | **11** | 81981 | 109813 | 21217 | 10.7 | 5979 | 21562 | 6.2 | 22.2 |
|  | **6** | 94618 | 128236 | 33036 | 11 | 8512 | 25381 | 7.7 | 22.9 |
|  | **9** | 125103 | 181452 | 48777 | 11.4 | 10138 | 45311 | 7 | 31.1 |
|  | **10** | 131105 | 183266 | 72886 | 11.5 | 17193 | 41819 | 13.1 | 31.9 |
|  | **16** | 131505 | 192818 | 57058 | 11.5 | 9987 | 51009 | 6.7 | 34.3 |
|  |  |  |  |  |  |  |  |  |  |
| **Including gene families with >10 hits** | **15** | 36573 | 50483 | 18653 | 10.2 | 0 | 6368 | 0 | 12.4 |
|  | **14** | 27744 | 37002 | 8813 | 9.7 | 0 | 4622 | 0 | 11.8 |
|  | **11** | 32443 | 42976 | 12108 | 10 | 0 | 4266 | 0 | 9.5 |
|  | **6** | 29344 | 36353 | 13384 | 9.9 | 0 | 4914 | 0 | 13.1 |
|  | **9** | 40187 | 54842 | 18383 | 10.3 | 0 | 6871 | 0 | 12.9 |
|  | **10** | 35359 | 42418 | 18756 | 10.2 | 0 | 10141 | 0 | 27.1 |
|  | **16** | 41216 | 55372 | 20253 | 10.3 | 0 | 7958 | 0 | 15 |
|  | ***Numbers of observed genes are slightly different than in the diagram Fig. 5. This is method related, as iterated subsampling was used to determine the indices listed in this table. Overall trends are similar in both approaches.**  ****SSGabs: Absolute single sequence gene – a gene represented by only one read in the whole dataset**  ****SSGrel: Relative single sequence gene – a gene represented by only one read in one sample, but more reads in other samples. A concept analogous to singleton operational taxonomic units SSOabs and SSOrel** [14] | | | | | | | | |

**Table S6: Percentage of shared gene families between HMMV metagenomes**

| **Samples** | **15** | **14** | **11** | **9** | **6** | **10** | **16** |
| --- | --- | --- | --- | --- | --- | --- | --- |
| **15** | 100 | 60 | 66 | 48 | 30 | 41 | 48 |
| **14** | 60 | 100 | 77 | 31 | 18 | 27 | 30 |
| **11** | 66 | 77 | 100 | 35 | 20 | 29 | 35 |
| **9** | 48 | 31 | 35 | 100 | 60 | 54 | 79 |
| **6** | 30 | 18 | 20 | 60 | 100 | 52 | 58 |
| **10** | 41 | 27 | 29 | 54 | 52 | 100 | 56 |
| **16** | 48 | 30 | 35 | 79 | 58 | 56 | 100 |

**Supplementary References**

1. Ruff SE, Felden J, Marcon Y, Ramette A, Boetius A. Development of bacterial and archaeal communities in erupted subsurface muds at the Håkon Mosby mud volcano. 2016; https://doi.pangaea.de/10.1594/PANGAEA.861266.

2. Marcon Y, Sahling H, Bohrmann G. LAPM: a tool for underwater large-area photo-mosaicking. *Geosci Instrumentation, Methods Data Syst* 2013; **2**: 189–198.

3. Marcon Y. Georeferenced photomosaic of the Håkon Mosby mud volcano during Maria S. Merian cruise MSM16/2 (LOOME), link to GeoTIFF archive (32 GB). 2016; https://doi.org/10.1594/PANGAEA.864702.

4. Cline JD. Spectrophotometric determination of hydrogen sulfide in natural waters. *Limnol Oceanogr* 1969; **14**: 454–458.

5. Hall POJ, Aller RC. Rapid, Small-Volume, Flow Injection Analysis for ΣCO2 and NH4+ in Marine and Freshwaters. *Limnol Oceanogr* 1992; **37**: 1113–1119.

6. Lustwerk RL, Burdige DJ. Elimination of Dissolved Sulfide Interference in the Flow Injection Determination of by Addition of Molybdate. *Limnol Oceanogr* 1995; 1011–1012.

7. Grasshoff K, Kremling K, Ehrhardt M. Methods of seawater analysis, 3rd ed. 1999. WILEY-VCH, Weinheim, Germany.

8. Jørgensen BB. A comparison of methods for the quantification of bacterial sulfate reduction in coastal marine sediments. *Geomicrobiol J* 1978; **1**: 11–27.

9. Treude T, Boetius A, Knittel K, Wallmann K, Jørgensen BB. Anaerobic oxidation of methane above gas hydrates at Hydrate Ridge, NE Pacific Ocean. *Mar Ecol Prog Ser* 2003; **264**: 1–14.

10. Kallmeyer J, Ferdelman TG, Weber A, Fossing H, Jørgensen BB. A cold chromium distillation procedure for radiolabeled sulfide applied to sulfate reduction measurements. *Limnol Oceanogr Methods* 2004; **2**: 171–180.

11. Quast C, Pruesse E, Yilmaz P, Gerken J, Schweer T, Yarza P. The SILVA ribosomal RNA gene database project: improved data processing and web-based tools. *Nucleic Acids Res* 2013; **41**.

12. Ruff SE, Ramette A, Boetius A. Metadata und statistical analysis of archaeal and bacterial sequences originating from sediments of the Håkon Mosby mud volcano. 2016; https://doi.pangaea.de/10.1594/PANGAEA.861873.

13. Gower JC. Generalized Procrustes analysis. *Psychometrika* 1975; **40**: 33–51.

14. Gobet A, Böer SI, Huse SM, van Beusekom JEE, Quince C, Sogin ML, et al. Diversity and dynamics of rare and of resident bacterial populations in coastal sands. *ISME J* 2012; **6**: 542–553.

15. Ludwig W, Strunk O, Westram R, Richter L, Meier H, Yadhukumar, et al. ARB: a software environment for sequence data. *Nucleic Acids Res* 2004; **32**: 1363–1371.

16. Bushnell B, Rood J, Singer E. BBMerge – Accurate paired shotgun read merging via overlap. *PLoS One* 2017; **12**: e0185056.

17. Abubucker S, Segata N, Goll J, Schubert AM, Izard J, Cantarel BL, et al. Metabolic Reconstruction for Metagenomic Data and Its Application to the Human Microbiome. *PLOS Comput Biol* 2012; **8**: e1002358.

18. R Core Team. R: A language and environment for statistical computing. 2018. R Foundation for Statistical Computing, Vienna, Austria.

19. Langmead B, Salzberg SL. Fast gapped-read alignment with Bowtie 2. *Nat Methods* 2012; **9**: 357.

20. Buchfink B, Xie C, Huson DH. Fast and sensitive protein alignment using DIAMOND. *Nat Methods* 2014; **12**: 59.

21. Suzek BE, Wang Y, Huang H, McGarvey PB, Wu CH, Consortium the U. UniRef clusters: a comprehensive and scalable alternative for improving sequence similarity searches. *Bioinformatics* 2015; **31**: 926–932.

22. Oksanen J, Blanchet FG, Kindt R, Legendre P, Minchin PR, O´Hara RB, et al. vegan: Community Ecology Package. 2012.

23. Maechler M, Rousseeuw P, Struyf A, Hubert M, Hornik K. cluster: Cluster Analysis Basics and Extensions. 2018.

24. Conway JR, Lex A, Gehlenborg N. UpSetR: An R Package For The Visualization Of Intersecting Sets And Their Properties. *bioRxiv* 2017.

25. Wickham H. ggplot2: Elegant Graphics for Data Analysis. 2009. Springer-Verlag New York, New York.

26. Milkov A V, Vogt PR, Crane K, Lein AY, Sassen R, Cherkashev GA. Geological, geochemical, and microbial processes at the hydrate-bearing Haakon Mosby mud volcano: a review. *Chem Geol* 2004; **205**: 347–366.

27. Niemann H, Lösekann T, de Beer D, Elvert M, Nadalig T, Knittel K, et al. Novel microbial communities of the Haakon Mosby mud volcano and their role as a methane sink. *Nature* 2006; **443**: 854–858.

28. Felden J, Wenzhöfer F, Feseker T, Boetius A. Transport and consumption of oxygen and methane in different habitats of the Håkon Mosby Mud Volcano (HMMV). *Limnol Oceanogr* 2010; **55**: 2366–2380.

29. Feseker T, Boetius A, Wenzhöfer F, Blandin J, Olu K, Yoerger DR, et al. Eruption of a deep-sea mud volcano triggers rapid sediment movement. *Nat Commun* 2014; **5**.

30. Feseker T, Foucher JP, Harmegnies F. Fluid flow or mud eruptions? Sediment temperature distributions on Håkon Mosby mud volcano, SW Barents Sea slope. *Mar Geol* 2008; **247**: 194–207.

31. Foucher J-P, Dupré S, Scalabrin C, Feseker T, Harmegnies F, Nouzé H. Changes in seabed morphology, mud temperature and free gas venting at the Håkon Mosby mud volcano, offshore northern Norway, over the time period 2003 - 2006. *Geo-Marine Lett* 2010; **30**: 157–167.

32. De Beer D, Sauter E, Niemann H, Kaul N, Foucher J-P, Witte U, et al. In situ Fluxes and Zonation of Microbial Activity in Surface Sediments of the Håkon Mosby Mud Volcano. *Limnol Oceanogr* 2006; **51**: 1315–1331.

33. Felden J, Wenzhöfer F, Boetius A. Methane and sulphate consumption in different habitats of the Håkon Mosby Mud Volcano (HMMV). *Suppl to Felden al (2010) Limnol Oceanogr 55(6), 2366-2380, doi104319/* . 2010. PANGAEA.

34. Feseker T, Boetius A, Wenzhöfer F, Blandin J, Olu K, Yoerger D, et al. Eruption of the Håkon Mosby mud volcano recorded by the long-term observatory on mud-volcano eruptions (LOOME) between 2009 and 2010. *Suppl to Feseker, T al Erupt a Deep mud volcano triggers rapid sediment movement Nat Commun 5, 5385, https//doi.org/101038/ncomms6385* 2014; https://doi.org/10.1594/PANGAEA.830324.

35. Yarza P, Yilmaz P, Pruesse E, Glöckner FO, Ludwig W, Schleifer K-H, et al. Uniting the classification of cultured and uncultured bacteria and archaea using 16S rRNA gene sequences. *Nat Rev Microbiol* 2014; **12**: 635–645.

36. Ruff SE, Ramette A, Boetius A. Relative abundance of prokaryotes in sediments of the Håkon Mosby mud volcano. 2016; https://doi.pangaea.de/10.1594/PANGAEA.861872.

37. Lösekann T, Knittel K, Nadalig T, Fuchs B, Niemann H, Boetius A, et al. Diversity and Abundance of Aerobic and Anaerobic Methane Oxidizers at the Haakon Mosby Mud Volcano, Barents Sea. *Appl Environ Microbiol* 2007; **73**: 3348–3362.

38. Stahl DA, Amann R. Nucleic acid techniques in bacterial systematics. In: Stackebrandt E, Goodfellow M (eds).1991. John Wiley & Sons Ltd., Chichester, England, pp 205–248.

39. Manz W, Eisenbrecher M, Neu TR, Szewzyk U. Abundance and spatial organization of Gram-negative sulfate-reducing bacteria in activated sludge investigated by in situ probing with specific 16S rRNA targeted oligonucleotides. *FEMS Microbiol Ecol* 1998; **25**: 43–61.

40. Amann RI, Binder BJ, Olson RJ, Chisholm SW, Devereux R, Stahl DA. Combination of 16S rRNA-targeted oligonucleotide probes with flow cytometry for analyzing mixed microbial populations. *Appl Environ Microbiol* 1990; **56**: 1919–1925.

41. Daims H, Brühl A, Amann R, Schleifer KH, Wagner M. The domain-specific probe EUB338 is insufficient for the detection of all Bacteria: development and evaluation of a more comprehensive probe set. *Syst Appl Microbiol* 1999; **22**: 434–444.

42. Boetius A, Ravenschlag K, Schubert CJ, Rickert D, Widdel F, Gieseke A, et al. A marine microbial consortium apparently mediating anaerobic oxidation of methane. *Nature* 2000; **407**: 623–626.

43. Ruff SE, Arnds J, Knittel K, Amann R, Wegener G, Ramette A, et al. Microbial Communities of Deep-Sea Methane Seeps at Hikurangi Continental Margin (New Zealand). *PLoS One* 2013; **8**: e72627.

44. Wallner G, Amann R, Beisker W. Optimizing fluorescent in situ hybridization with rRNA-targeted oligonucleotide probes for flow cytometric identification of microorganisms. *Cytometry* 1993; **14**: 136–43.
